# NCOA2 promotes lytic reactivation of Kaposi’s sarcoma-associated herpesvirus by enhancing the expression of the master switch protein RTA

**DOI:** 10.1371/journal.ppat.1008160

**Published:** 2019-11-21

**Authors:** Xiaoqin Wei, Lei Bai, Lianghui Dong, Huimei Liu, Peidong Xing, Zhiyao Zhou, Shuwen Wu, Ke Lan

**Affiliations:** 1 State Key Laboratory of Virology, College of Life Sciences, Wuhan University, Wuhan, China; 2 University College London, Gower Street, London, United Kingdom; Wistar Institute, UNITED STATES

## Abstract

Reactivation of Kaposi’s sarcoma-associated herpesvirus (KSHV) is important for persistent infection in the host as well as viral oncogenesis. The replication and transcription activator (RTA) encoded by KSHV ORF50 plays a central role in the switch from viral latency to lytic replication. Given that RTA is a transcriptional activator and RTA expression is sufficient to activate complete lytic replication, RTA must possess an elaborate mechanism for regulating its protein abundance. Previous studies have demonstrated that RTA could be degraded through the ubiquitin-proteasome pathway. A protein abundance regulatory signal (PARS), which consists of PARS I and PARS II, at the C-terminal region of RTA modulates its protein abundance. In the present study, we identified a host protein named Nuclear receptor coactivator 2 (NCOA2), which can interact with RTA *in vitro* and *in vivo*. We further showed that NCOA2 binds to the PARS II domain of RTA. We demonstrated that NCOA2 enhances RTA stability and prevents the proteasome-mediated degradation of RTA by competing with MDM2, an E3 ubiquitin ligase of RTA that interacts with the PARS II domain. Moreover, overexpression of NCOA2 in KSHV-infected cells significantly enhanced the expression level of RTA, which promotes the expression of RTA downstream viral lytic genes and lytic replication. In contrast, silencing of endogenous NCOA2 downregulated the expression of viral lytic genes and impaired viral lytic replication. Interestingly, we also found that RTA upregulates the expression of NCOA2 during lytic reactivation. Taken together, our data support the conclusion that NCOA2 is a novel RTA-binding protein that promotes RTA-driven lytic reactivation by increasing the stability of RTA, and the RTA-NCOA2 positive feedback regulatory loop plays an important role in KSHV reactivation.

## Author summary

Reactivation of KSHV from latency to lytic replication plays an important role in viral spread, establishment of lifelong latent infection and disease progression. RTA, the lytic switch protein, is essential and sufficient for triggering the full viral lytic program. Here, we report a host protein named NCOA2 as a novel RTA-binding protein. Direct interaction of NCOA2 with RTA increased the expression level of RTA. Further study revealed that NCOA2 competes with the E3 ubiquitin ligase of RTA, MDM2, to interact with the PARS II domain of RTA, which inhibits RTA degradation and enhances the stability of RTA. In the context of KSHV-infected cells, we showed that NCOA2 plays an important role in promoting RTA-driven lytic reactivation.

## Introduction

Kaposi’s sarcoma-associated herpesvirus (KSHV), also known as human herpesvirus 8, is an oncogenic γ herpesvirus closely associated with an endothelial neoplasm, Kaposi’s sarcoma (KS), and two other lymphoproliferative diseases, primary effusion lymphoma (PEL) and multicentric Castleman’s disease (MCD) [1–3]. Similar to other herpesviruses, KSHV has two types of life cycles: latency and lytic replication [4]. KSHV evades host immunity by establishing a lifelong latent infection. In this phase, KSHV exhibits highly restricted gene expression patterns, and few viral particles are produced [5, 6]. Although latent infection is the default life cycle of KSHV, the virus can be reactivated from latency by physiological stimuli, leading to the production of mature viruses [7–10]. Lytic replication promotes the recruitment of new cells to latency to replace those that have segregated the viral episome, suggesting an important role for lytic replication in persistent viral infection [11–14]. HIV/AIDS patients receiving antiherpetic drugs to block lytic replication showed a reduced incidence of KS, confirming the role of lytic DNA replication and gene products in KSHV-mediated tumorigenesis [15–17]. Thus, identifying the molecular events during the lytic replication cycle is critical to understanding KSHV pathogenesis and developing promising strategies to disrupt persistent KSHV infection and prevent the occurrence of KSHV-related diseases.

The switch of KSHV from latency to the lytic cycle is mainly regulated by replication and transcription activator (RTA), which is encoded by KSHV ORF50 [18–22]. RTA is a multifunctional protein that is involved in the assembly of KSHV ori-Lyt replication complexes by recruiting specific proteins required for lytic DNA synthesis [23] and also functions as a transcriptional activator. RTA activates a number of downstream viral and cellular target genes by at least three mechanisms, which have been well studied, including directly binding to specific motifs in some viral promoters, such as PAN and K12 [24]; indirectly accessing certain promoters of target genes through cooperating with various host transcription factors, such as RBP-Jκ, Oct-1, C/EBPα, and Sp1 [22, 25–29]; or serving as an intrinsic E3 ubiquitin ligase to promote the degradation of host transcription repressors, such as K-RBP and IRF-7 [30–33]. Ectopic expression of RTA is sufficient to disrupt KSHV latency and drive a complete lytic replication cycle [20]. Thus, the abundance of RTA protein must be tightly regulated to facilitate life-long persistent infection of KSHV.

Previous studies showed that RTA is polyubiquitinated, and its abundance is controlled by the proteasomal degradation pathway. MDM2, a human oncoprotein [34], has been identified as an E3 ligase of RTA that interacts with RTA to promote the degradation of RTA in host cells [35]. An abundance regulatory signal [protein abundant regulatory signal (PARS)] domain within the C-terminal region of RTA plays a critical role in regulating the abundance of RTA. PARS is composed of two subcomponents, PARS I and PARS II. PARS I is located between amino acids (aa) 490 and 535, whereas PARS II is located between aa 590 and 650. Mutation or deletion of either component enhanced RTA expression [36]. The PARS I domain, as a nuclear localization signal (NLS), mediates the translocation of RTA from the cytoplasm to the nucleus. Meanwhile, PARS II serves as a docking site for ubiquitin enzymes to control the degradation of RTA in the nucleus. The MDM2 protein binds to the PARS domain to promote RTA degradation through the proteasomal-dependent degradation pathway [35]. A previous study showed that viral small peptide 1 (vSP1), an immediate-early gene product encoded by the T3.0 transcript of KSHV, increases the stability of RTA by binding to the PARS II domain and blocks the ubiquitination and degradation of RTA [37]. Thus, the abundance of RTA is tightly controlled by cellular and viral molecules. However, how the stability of RTA is finely regulated in cells and whether there are other host proteins involved in this process have not been fully elucidated.

Despite extensive studies on how RTA activates its promoter [21] and its downstream viral promoters [38], analyses of the regulatory mechanisms of RTA protein level are lacking. In this study, NCOA2 was identified as a novel RTA-binding protein. We found that NCOA2 strongly binds to the PARS II domain of RTA and upregulates RTA expression. We further showed that NCOA2 blocks the E3 ubiquitin enzyme MDM2 from binding to the PARS domain of RTA, which protects RTA from ubiquitin-proteasome-mediated degradation during the viral lytic reactivation process. We demonstrated that ectopic expression of NCOA2 substantially enhanced viral lytic replication, whereas knockdown or knockout of NCOA2 largely impaired the ability of RTA to drive lytic replication. Interestingly, we also found that NCOA2 expression was upregulated upon RTA activation during the viral lytic cycle, suggesting a positive feedback regulatory loop between NCOA2 and RTA, which is important to further enhance RTA expression. These results demonstrated that NCOA2 plays an important role in regulating RTA stability and therefore facilitates KSHV lytic replication mediated by RTA.

## Results

### NCOA2 interacts with KSHV RTA

**Fig 1:**
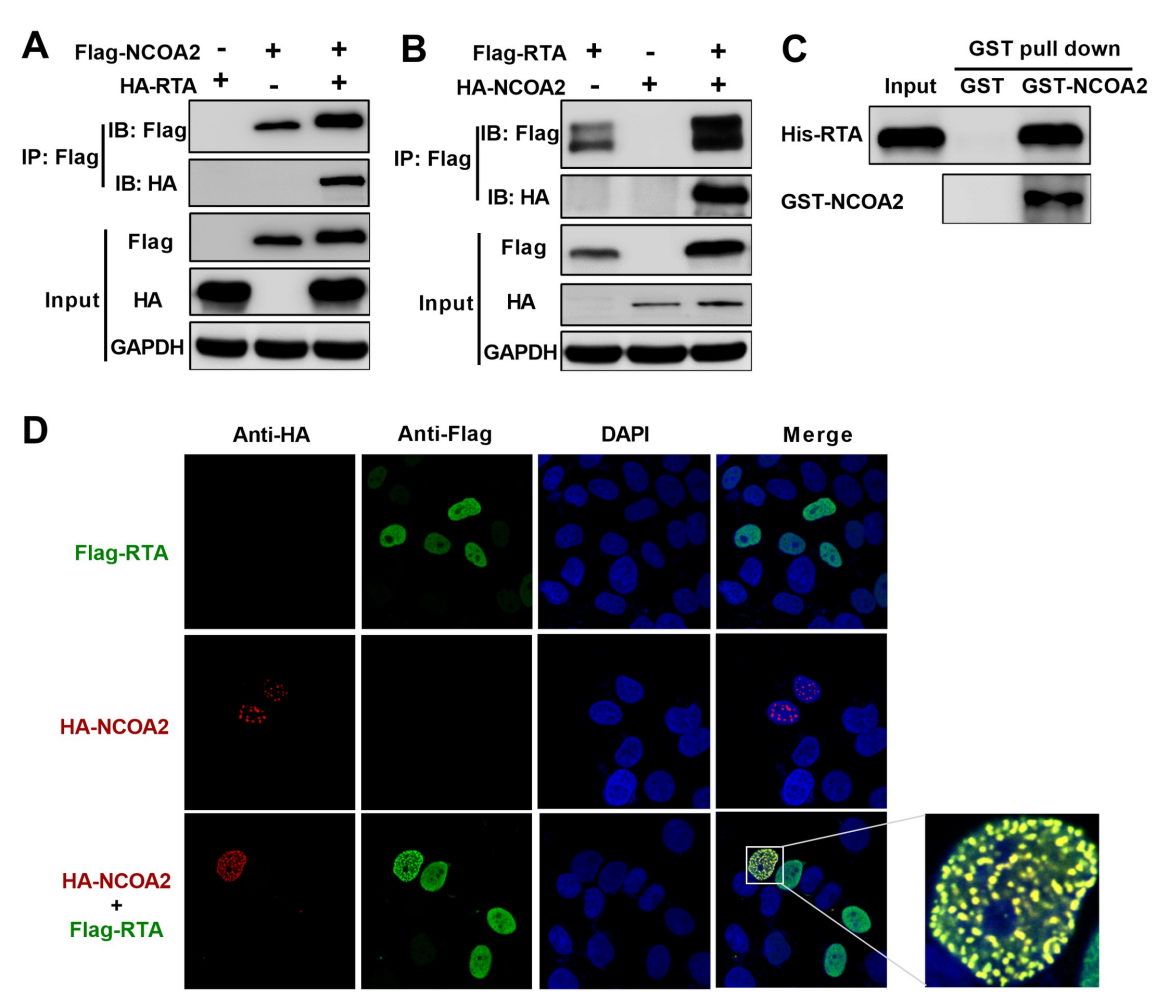
NCOA2 interacts with KSHV RTA. (A) First, 293T cells were transfected with Flag-NCOA2 alone or HA-RTA alone or cotransfected with Flag-NCOA2 along with HA-RTA. Cell lysates were immunoprecipitated with anti-Flag antibody and then analyzed by western blotting with the indicated antibodies. (B) The 293T cells were transfected with Flag-RTA alone or HA-NCOA alone or cotransfected with Flag-RTA along with HA-NCOA2. Cell lysates were immunoprecipitated with anti-Flag antibody and then analyzed by western blotting with the indicated antibodies. (C) *In vitro* GST affinity binding assay. Bacterially expressed GST alone and GST-NCOA2 attached to GST-Sepharose beads were incubated with the purified His-tagged RTA, and the pull-down lysates were immunoblotted with anti-His or anti-GST antibodies. (D) Colocalization of NCOA2 and RTA in HeLa cells. Following transfection with Flag-RTA and HA-NCOA2, HeLa cells were fixed with 4% paraformaldehyde and then stained with anti-HA and anti-Flag antibodies. Secondary antibodies conjugated to FITC or Cy3 were used to visualize the stained RTA and NCOA proteins, respectively. Diamidino-2-phenylindole shows the nuclei of cells.

To further understand the mechanism by which RTA functions are regulated, we used the Flag-tag-based affinity purification method to identify novel host factors interacting with RTA. RTA was overexpressed in HEK293T cell lines and HEK293T.219 cell lines to seek the RTA-interacting proteins. We immunoprecipitated the proteins interacted with RTA and analyzed the proteins by mass spectrometry coupled with bioinformatics methods (S1A Fig). Based on the mass spectrometry data, we constructed the Venn diagram identifying 77 RTA interacting-proteins enriched both in HEK293T cell lines and HEK293T.219 cells (S1B Fig). The results are summarized in S1 Table. A host protein named NCOA2 was one of the candidate proteins that can potentially interact with RTA. The interaction between RTA and NCOA2 was confirmed by coimmunoprecipitation (Co-IP) assays. As shown in Fig 1A, 293T cells were transfected with Flag-tagged NCOA2 and HA-tagged RTA individually or together. RTA was coimmunoprecipitated with NCOA2 (Fig 1A). The reverse Co-IP experiment showed that the NCOA2 protein was specifically coimmunoprecipitated with RTA (Fig 1B). To determine whether this interaction was direct, we investigated the interaction by an *in vitro* binding assay. *In vitro*-purified His-fused RTA was incubated with purified GST or GST-fused NCOA2 beads. The results showed that NCOA2 directly interacts with RTA (Fig 1C).

To corroborate the above results from the immunoprecipitation and *in vitro* binding assays, we further performed immunofluorescence assays to determine whether NCOA2 and RTA could be colocalized in the same cellular compartment. HeLa cells were cotransfected transiently with Flag-tagged RTA and HA-tagged NCOA2. RTA and NCOA2 were colocalized to the same nuclear compartment in HeLa cells (Fig 1D). This result suggested that exogenously transfected NCOA2 and RTA proteins colocalized in the nucleus.

**Fig 2:**
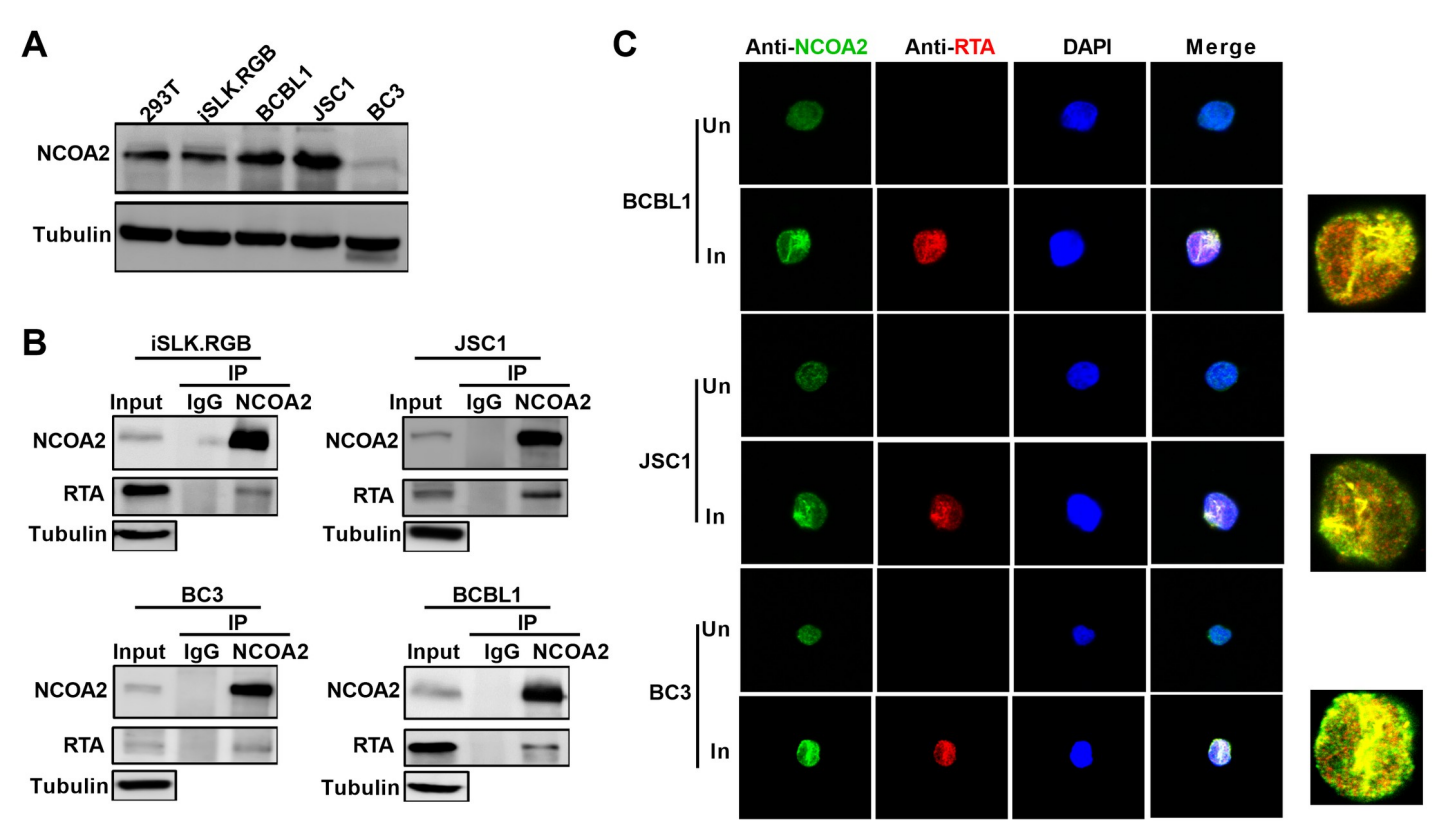
The interaction between endogenous NCOA2 and RTA. (A) NCOA2 expression in HEK293T cells and KSHV-positive human cells (iSLK.RGB, BCBL1, JSC1 and BC3) was detected by western blotting. (B) Co-IP of endogenous RTA and NCOA2 in KSHV-positive cells. Lytic replication of KSHV in these cells was induced by dox or VPA, and cell lysates were subjected to immunoprecipitation with anti-NCOA2 antibody or rabbit IgG controls. Purified proteins, along with input samples, were detected by western blotting with the indicated antibodies. (C) Endogenous NCOA2 colocalizes with endogenous RTA in the nucleus. KSHV-positive B cells that were uninduced (Un) or induced with VPA (In) were fixed and stained with anti-NCOA2 antibody and anti-RTA antibody, followed by incubation with secondary antibodies conjugated to FITC or Cy3. The right sides show a high magnified view.

To confirm the interaction between endogenous NCOA2 and RTA, we first examined the expression levels of NCOA2 in different cell lines. Western blotting analysis showed that NCOA2 is expressed in 293T cells and several KSHV latently infected cell lines (Fig 2A). We then carried out Co-IP with KSHV-infected cells (iSLK.RGB, BCBL1, JSC1, BC3) that harbored latent KSHV episomes. After these KSHV-infected cells were induced by doxycycline (dox) (iSLK.RGB) or treated with valproic acid (VPA) (BCBL1, JSC1 and BC3), which is an inducer of KSHV lytic replication [39], for 24 hours (h) to activate the expression of endogenous RTA, cell lysates were immunoprecipitated with anti-NCOA2 antibody or rabbit IgG control. As expected, RTA was associated with the endogenous NCOA2 protein in KSHV-infected cells (Fig 2B). We also performed immunofluorescence assays to explore whether endogenous NCOA2 and RTA could be colocalized in similar nuclear compartments in naturally KSHV-infected BCBL1, BC3 and JSC1 cells. Twelve hours after VPA induction, cells were fixed for immunofluorescence and probed with RTA as well as NCOA2 antibodies, followed by incubation with appropriate secondary antibodies. The results demonstrated that endogenous NCOA2 and RTA were colocalized in the same nuclear compartments of BCBL1, BC3 and JSC1 cells (Fig 2C). Taken together, these results indicated that the host NCOA2 protein is a novel KSHV RTA-interacting protein.

### Mapping the interaction domains in RTA and NCOA2

**Fig 3:**
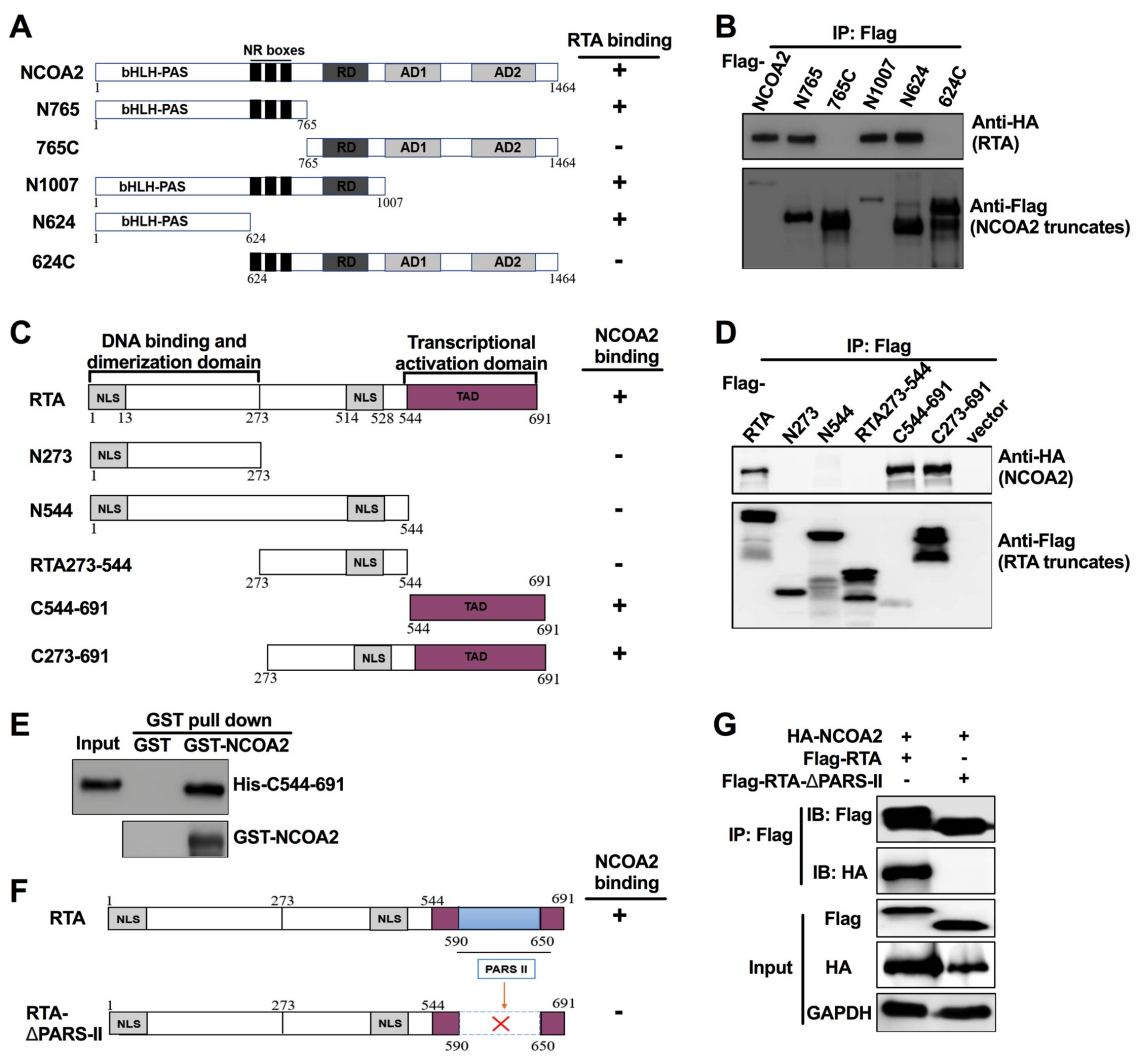
Mapping the interaction domains in RTA and NCOA2. (A) Truncated versions of NCOA2 are shown schematically, including N765 (1 aa-765 aa), 765C (765 aa-1464 aa), N1007 (1 aa-1007 aa), N624 (1 aa-624 aa), 624C (624 aa-1464 aa). The N-terminal bHLH-PAS domain, the centrally located nuclear receptor (NR) boxes responsible for NR binding, the C-terminal activation domain (AD1 and AD2) and a repression domain (RD) are marked. (B) Defining the RTA-interacting domain of NCOA2. Co-IP and western blotting of 293T cells transfected with HA-tagged RTA along with a vector expressing the indicated Flag-tagged NCOA2 truncations or the full-length NCOA2. (C) Schematic diagram of the RTA protein and its deletion mutants, including N273 (1 aa-273 aa), N544 (1 aa-544 aa), RTA273-544 (273 aa-544 aa), C544-691 (544 aa-691 aa), C273-691 (273 aa-691 aa). The nuclear localization (NLS), DNA binding and dimerization and transcriptional activation domain (TAD) are marked. (D) Defining the NCOA2-interacting domain of RTA. Co-IP and western blotting of 293T cells transfected with HA-tagged NCOA2 along with Flag-tagged RTA truncations or full-length RTA. An empty vector was used as a negative control. (E) *In vitro* GST affinity binding assay. Bacterially expressed GST alone and GST-NCOA2 attached to GST-Sepharose beads were incubated with the purified His-tagged RTA mutation (His-C544-691). The pull-down lysates were immunoblotted with anti-His or anti-GST antibody. (F) Schematic diagram of a PARS II region deletion mutant of RTA. (G) Co-IP and western blotting of 293T cells transfected with HA-NCOA2 along with Flag-tagged PARS II region deletion mutants.

NCOA2 is a modular protein with well-defined protein-protein interaction domains, including the N-terminal bHLH-PAS domain, the centrally located nuclear receptor (NR) boxes responsible for NR binding, the C-terminal activation domain (AD) and a repression domain (RD) (Fig 3A) [40]. To evaluate the importance of individual NCOA2 regions responsible for the interaction with RTA, we generated a series of Flag-tagged NCOA2 deletion mutants (Fig 3A). Then, 293T cells were cotransfected with RTA and wild-type NCOA2 or NCOA2 mutants. The Co-IP assay showed that the N624 mutant (from 1 aa to 624 aa), which contains the bHLH-PAS domain of NCOA2, had the crucial region responsible for the association of NCOA2 with RTA (Fig 3B).

A similar approach was used to define the RTA region required for interaction with NCOA2. A series of truncated versions of Flag-tagged RTA were generated and examined for their ability to interact with NCOA2 (Fig 3C). We first determined the cellular localization of these RTA mutants and found all mutants were located in the nucleus (S2 Fig), with C544-691 (from 544 aa to 691 aa) and N273 (from 1 aa to 273 aa) primarily localized in the nucleus but also present in the cytoplasm. C544-691 currently has no known NLS, and we speculate that it might have other nuclear import pathways instead of the classical NLS signaling. As shown in Fig 3D, NCOA2 was coimmunoprecipitated by the RTA truncation that contains the transcriptional activation domain (TAD, from 544 aa to 691 aa). The GST pulldown assay showed that the interaction between the TAD of RTA and NCOA2 was direct (Fig 3E). The TAD contained a portion of the regulatory domain PARS, which consists of two components, namely, PARS I (490 aa–535 aa) and PARS II (590 aa–650 aa). Mutation or deletion of either component resulted in high RTA protein expression [36]. We further showed that RTA-△PARS-II, an RTA mutant in which the PARS II domain was deleted, lost the ability to interact with NCOA2 (Fig 3F and 3G). Thus, the PARS II domain of RTA is the minimum region responsible for the interaction with NCOA2.

### NCOA2 stabilizes RTA protein expression

**Fig 4:**
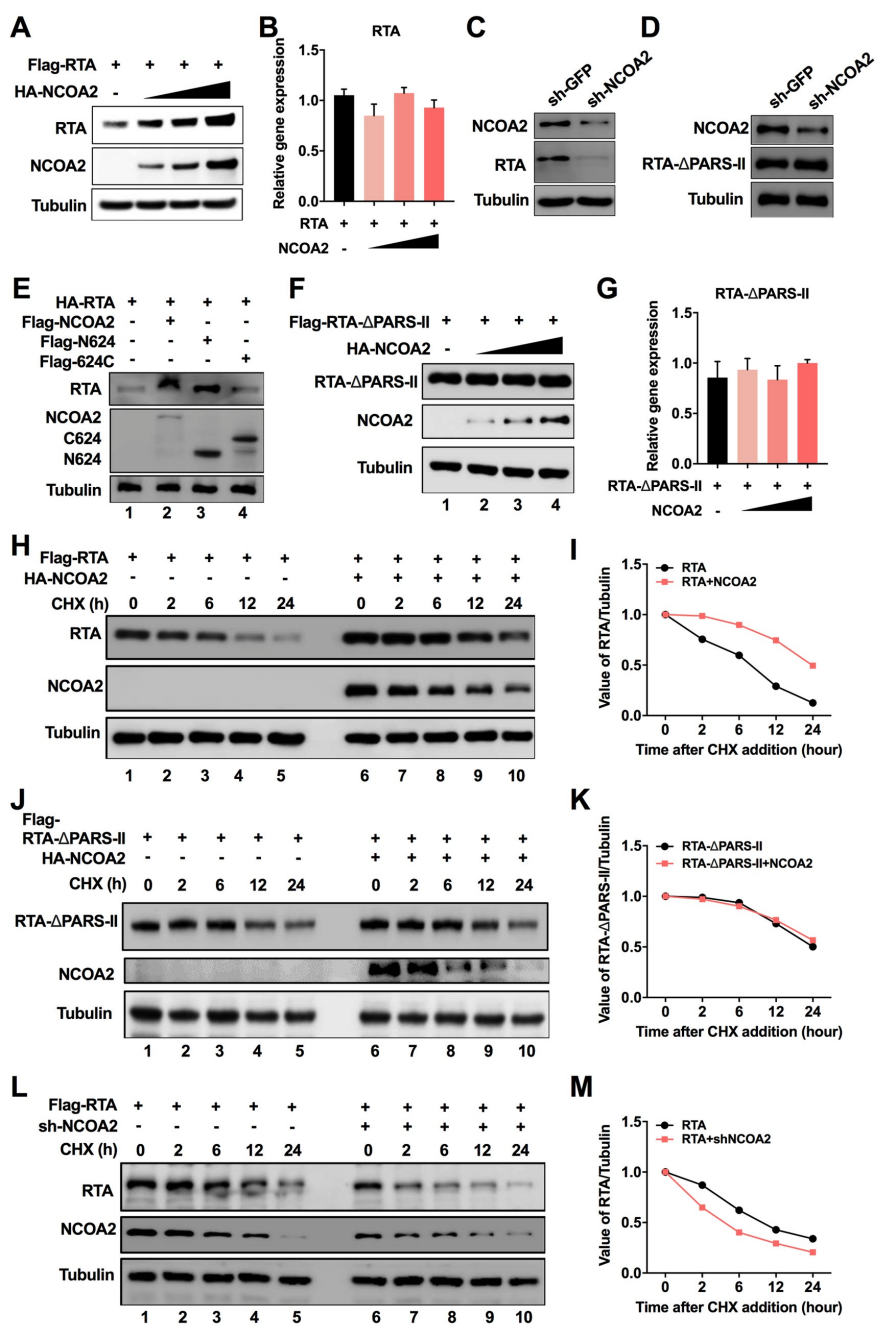
NCOA2 stabilizes RTA protein expression. (A) Effect of NCOA2 on RTA expression. First, 293T cells were cotransfected with 1 μg of RTA expression plasmid and increasing amounts of NCOA2 expression vector (0, 0.5, 1, 2 μg). The expression of RTA proteins was examined by immunoblotting with the indicated antibodies. (B) The 293T cells were treated as in (A). RTA mRNA was detected using RT-qPCR with the indicated primers. (C) shRNA-NCOA2 and shRNA-GFP plasmids were transfected into 293T cells for 12 h. Then, cells were transfected with the Flag-tagged RTA expression plasmid. The expression of NCOA2 and RTA was determined by immunoblotting with the indicated antibodies. (D) shRNA-NCOA2 and shRNA-GFP plasmids were transfected into 293T cells for 12 h. Then cells were transfected with the Flag-tagged RTA-△PARS-II expression plasmid. The expression of NCOA2 and RTA-△PARS-II was determined by immunoblotting with the indicated antibodies. (E) The N-terminal truncation of NCOA2 regulates the stability of RTA. First, 293T cells were cotransfected with HA-tagged RTA expression vector with or without NCOA2 and its truncations. Then, RTA expression levels were examined by western blotting using anti-HA antibodies. (F) Effect of NCOA2 on the expression of the RTA mutant with deletion of the PARS II region. First, 293T cells were cotransfected with 1 μg of Flag-tagged RTA-△PARS-II expression plasmid and increasing amounts of NCOA2 expression vector (0, 0.5, 1, 2 μg). Then, the expression of RTA-△PARS-II proteins was examined by immunoblotting with the indicated antibodies. (G) The 293T cells were treated as in (F), and the mRNA level of RTA-△PARS-II was detected using RT-qPCR with the indicated primers. (H) Measurement of RTA stability in the absence and presence of NCOA2. First, 293T cells were transfected with Flag-tagged RTA with or without the NCOA2 expression plasmid for 36 h. Then, cells were treated with 100 μg/ml of CHX and analyzed at different time points as indicated by immunoblotting for RTA. Tubulin was used as a control for equivalent sample loading. (I) The relative levels of RTA from immunoblots (H) were quantified by densitometry and normalized to the Tubulin level. The band intensities on the exposed film were plotted graphically. (J) Measurement of RTA-△PARS-II stability in the absence and presence of NCOA2. First, 293T cells were transfected with Flag-tagged RTA-△PARS-II with or without the NCOA2 expression plasmid for 36 h. Then, cells were treated with 100 μg/ml of CHX and analyzed at different time points as indicated by immunoblotting for RTA-△PARS-II. Tubulin was used as a control for equivalent sample loading. The band intensities on the exposed film are plotted graphically (K). (L) First, 293T cells were transfected with RTA expression plasmid with sh-NCOA2 or sh-GFP. Then, the cells were treated with 100 μg/ml of CHX and analyzed at different time points as indicated by immunoblotting for RTA. Tubulin was used as a control for equivalent sample loading. The band intensities on the exposed film were plotted graphically (M).

Previous studies reported that the degradation of RTA by ubiquitination involves both the PARS I and II domains. There are several lysines in PARS I that may function as ubiquitin acceptor sites, and PARS II serves as a dock site for ubiquitin E3 ligase [35]. Hence, we speculated that the binding of NCOA2 to the RTA PARS II motif may increase the stability of RTA by preventing its ubiquitination. To test this hypothesis, we coexpressed increasing amounts of NCOA2 with a constant amount of RTA in 293T cells, and the effect of NCOA2 on RTA expression was examined by western blotting analysis. The results showed that RTA expression was increased in the presence of NCOA2; moreover, RTA expression was enhanced by NCOA2 in a dose-dependent manner (Fig 4A). No significant changes were observed in RTA mRNA levels between the cells with or without expression of NCOA2 (Fig 4B). In contrast, knockdown of NCOA2 in 293T cells decreased the expression level of RTA in a transient transfection experiment (Fig 4C). However, knockdown of NCOA2 did not affect the expression level of RTA-△PARS-II, in which the PARS II domain was deleted from RTA and lost the binding activity with NCOA2 (Fig 4D). We also found that the truncation mutant N624 of NCOA2 maintained its ability to increase RTA expression, similar to the full-length NCOA2 (Fig 4E, lanes 2 and 3). However, the truncation mutant 624C of NCOA2 that cannot bind to RTA completely abolished the ability to enhance RTA expression (Fig 4E, lane 4). Additionally, the expression level of RTA-△PARS-II was no longer affected by NCOA2, and the mRNA levels of RTA-△PARS-II had no significant changes (Fig 4F and 4G). Based on these results, we conclude that the interaction between NCOA2 and the PARS II domain of RTA enhances the expression of RTA.

To further test if RTA stability was regulated by interacting with NCOA2, we determined the effect of NCOA2 on the half-life of RTA. RTA was transfected into 293T cells with or without the NCOA2 expression plasmid, and the cells were treated with cycloheximide (CHX) to block protein synthesis 24 h after transfection. We found that both the steady-state level and half-life of RTA were significantly increased in cells overexpressing NCOA2 compared to cells overexpressing the vector control (Fig 4H and 4I). However, NCOA2 expression had no effect on the half-life of the mutant RTA-△PARS-II that does not bind to NCOA2 (Fig 4J and 4K). Moreover, the expression level and the half-life of RTA were significantly reduced in NCOA2 knockdown cells (Fig 4L and 4M). These results suggested that NCOA2 enhances the stability of RTA through interacting with the PARS II domain and may abrogate the PARS-related regulation.

Previous study identified vSP1, a 48 aa peptide encoded in the T3.0 transcript antisense to RTA, also increased the stability of RTA by binding to the PARS II domain [37]. To better evaluate the role of NCOA2 and vSP1 in regulating RTA stability, we cotransfected 293T cells with NCOA2 and/or vSP1 with a constant amount of RTA expression plasmid. Indeed, both vSP1 and NCOA2 can increase the expression level of RTA respectively. Interestingly, there is no competitive binding of NCOA2 and vSP1 to RTA PARS-II domain, however, a slightly increased RTA expression was observed when NCOA2 and vSP1 were co-transfected compared with NCOA2 alone or vSP1 alone (S3A Fig). We also found that both vSP1 and NCOA2 can enhance the ability of each other to increase RTA expression in a dose-dependent manner (S3B and S3C Fig). The association between vSP1 and NCOA2 in controlling RTA levels remains further study.

### NCOA2 inhibits proteasome-mediated degradation of RTA

**Fig 5:**
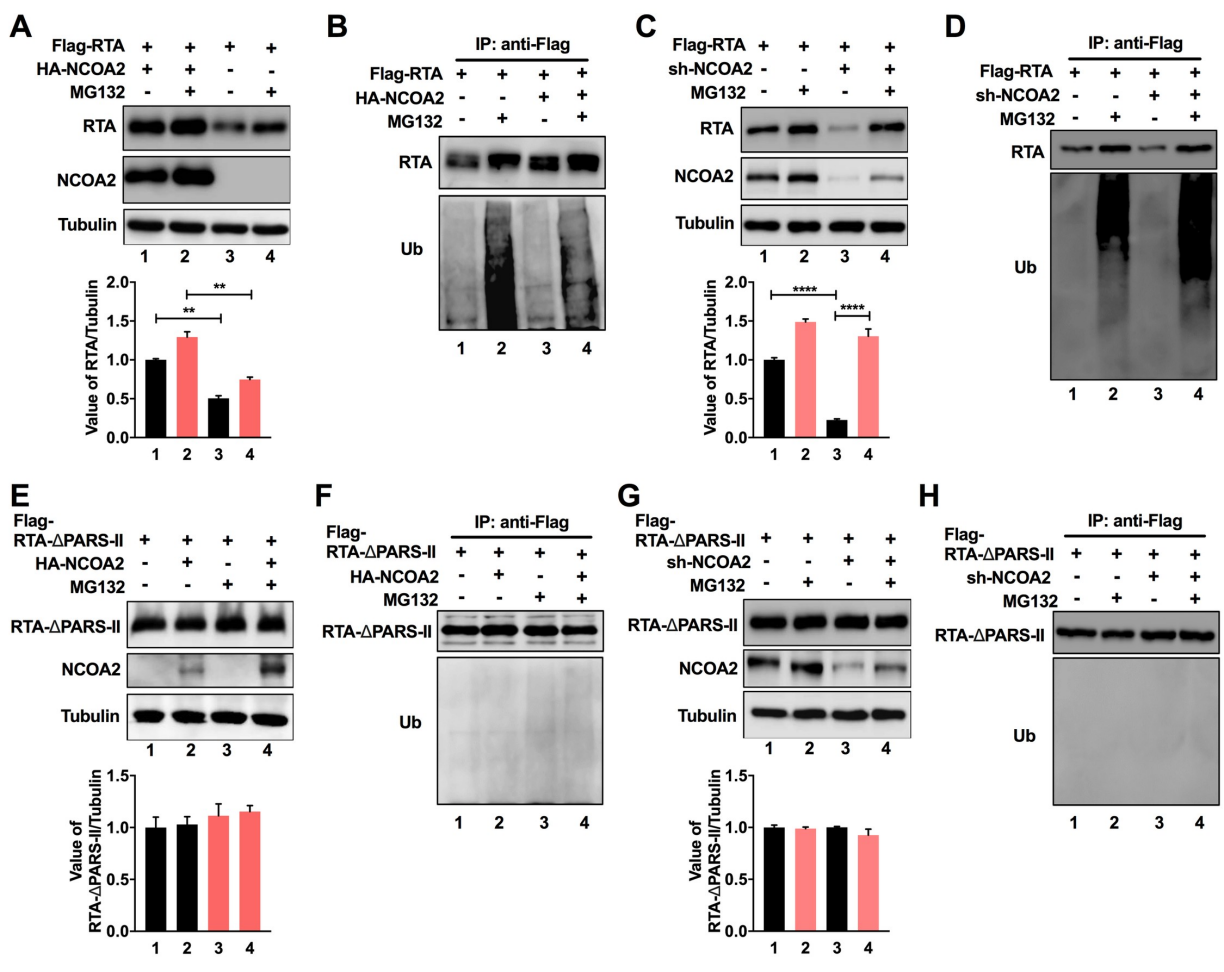
NCOA2 inhibits proteasome-mediated degradation of RTA. (A) NCOA2 inhibits the degradation of RTA. First, 293T cells were cotransfected with the indicated expression constructs for 36 h and then treated with 0.5 μM MG132 or 0.1% DMSO for another 6 h. The cells were lysed and used for western blotting with the indicated antibodies. The band intensities of RTA were plotted graphically. Data are from a minimum of three experimental replicates with a standard deviation. (B) NCOA2 inhibits the ubiquitination of RTA. The same lysates as in (A) were subjected to immunoprecipitation using anti-Flag antibody. Purified proteins, along with input samples, were analyzed by western blotting with anti-ubiquitin antibodies. (C) shRNA-NCOA2 and shRNA-GFP plasmids were transfected into 293T cells for 12 h. Then cells were transfected with the Flag-tagged RTA expression plasmid for 24 h and then treated as in (A). Cells were lysed and used for western blotting with the indicated antibodies. The band intensities of RTA were plotted graphically. Data were from a minimum of three experimental replicates with a standard deviation. (D) Same lysates as in (C) were subjected to immunoprecipitation using anti-Flag antibody, followed by western blotting with anti-ubiquitin antibodies. (E) First, 293T cells were cotransfected with NCOA2 and the RTA-△PARS-II mutant, and then, they were treated as in (A). The expression of the RTA-△PARS-II mutant was analyzed by western blotting. The band intensities of RTA were plotted graphically. (F) The same lysates as in (E) were subjected to immunoprecipitation using anti-Flag antibody, followed by western blotting with anti-ubiquitin antibodies. (G) 293T cells were transfected with shRNA-NCOA2 or shRNA-GFP plasmids, and then cells were transfected with RTA-△PARS-II mutant and treated as in (A). The expression of RTA-△PARS-II mutant was analyzed by western blotting. The band intensities of RTA were plotted graphically. (H) The same lysates as in (G) were subjected to immunoprecipitation using anti-Flag antibody, followed by western blotting with anti-ubiquitin antibodies. Statistical significance was analyzed with a two-tailed Student’s *t*-test (*P < 0.05 or **P < 0.01).

The PARS II domain serves as the binding site for E3 ligase to modulate the ubiquitination and degradation of RTA. Therefore, we hypothesize that the interaction between NCOA2 and the PARS II domain of RTA may block the binding of ubiquitin enzymes with the PARS II domain, which may consequently inhibit the proteasome-associated degradation of RTA and contribute to the stability of RTA. To test this hypothesis, we cotransfected 293T cells with plasmids that express NCOA2 and RTA, and 24 h post-transfection, the cells were treated with MG132. In the absence of NCOA2, the steady-state level of RTA was significantly increased in the cells exposed to MG132 (Fig 5A, lanes 3 and 4). However, in the presence of NCOA2, the abundance of RTA was dramatically enhanced regardless of the presence of MG132 (Fig 5A, lanes 1 and 2). To further study the effect of NCOA2 on RTA ubiquitination, we immunoprecipitated the cell lysates with an anti-Flag antibody that pulled down Flag-tagged RTA and performed western blotting with an anti-ubiquitin antibody. In the cells without ectopic expression of NCOA2 that were treated with MG132, we observed a high molecular weight poly-ubiquitin-RTA smear (Fig 5B, lane 2), which was significantly decreased in cells that expressed NCOA2 (Fig 5B, lane 4). Additionally, knockdown of NCOA2 significantly decreased expression level of RTA (Fig 5C, lane 3), which is rescued by treatment with MG132 (Fig 5C, lane 4). Poly-ubiquitination of RTA was elevated with the knockdown of NCOA2 expression when compared with the control (Fig 5D, lanes 2 and 4). These findings indicated that NCOA2 had a role in inhibiting ubiquitination of RTA. To test if the interaction between NCOA2 and RTA is necessary for NCOA2 inhibition of proteasome-mediated degradation of RTA, we also transfected 293T cells with plasmids that express NCOA2 and the mutant RTA-△PARS-II. We found that the RTA-△PARS-II protein was highly abundant in the cells, and MG132 treatment did not increase its expression level (Fig 5E, lanes 1 and 3), which suggested that the PARS II domain is involved in the proteasome-mediated degradation of RTA, similar to the results of previous studies [35]. Additionally, the abundance of the RTA-△PARS-II protein did not change in cells overexpressing NCOA2 (Fig 5E, lanes 2 and 4). Knockdown of NCOA2 had no effect on the expression level of RTA-△PARS-II (Fig 5G). Similar to a previous study, RTA was not ubiquitinated after the PARS II domain was deleted (Fig 5F and 5H). These results suggested that the PARS II domain regulates the proteasome-mediated degradation of RTA and that NCOA2 reduces ubiquitination of RTA by interacting with the PARS II domain of RTA. Notably, treatment of cells with MG132 also increased the abundance of NCOA2 (Fig 5A, 5C, 5E and 5G), suggesting that the stability of NCOA2 is also regulated by ubiquitin-proteasome pathway.

### NCOA2 inhibits the ubiquitination of RTA by blocking the interaction of RTA with MDM2

**Fig 6:**
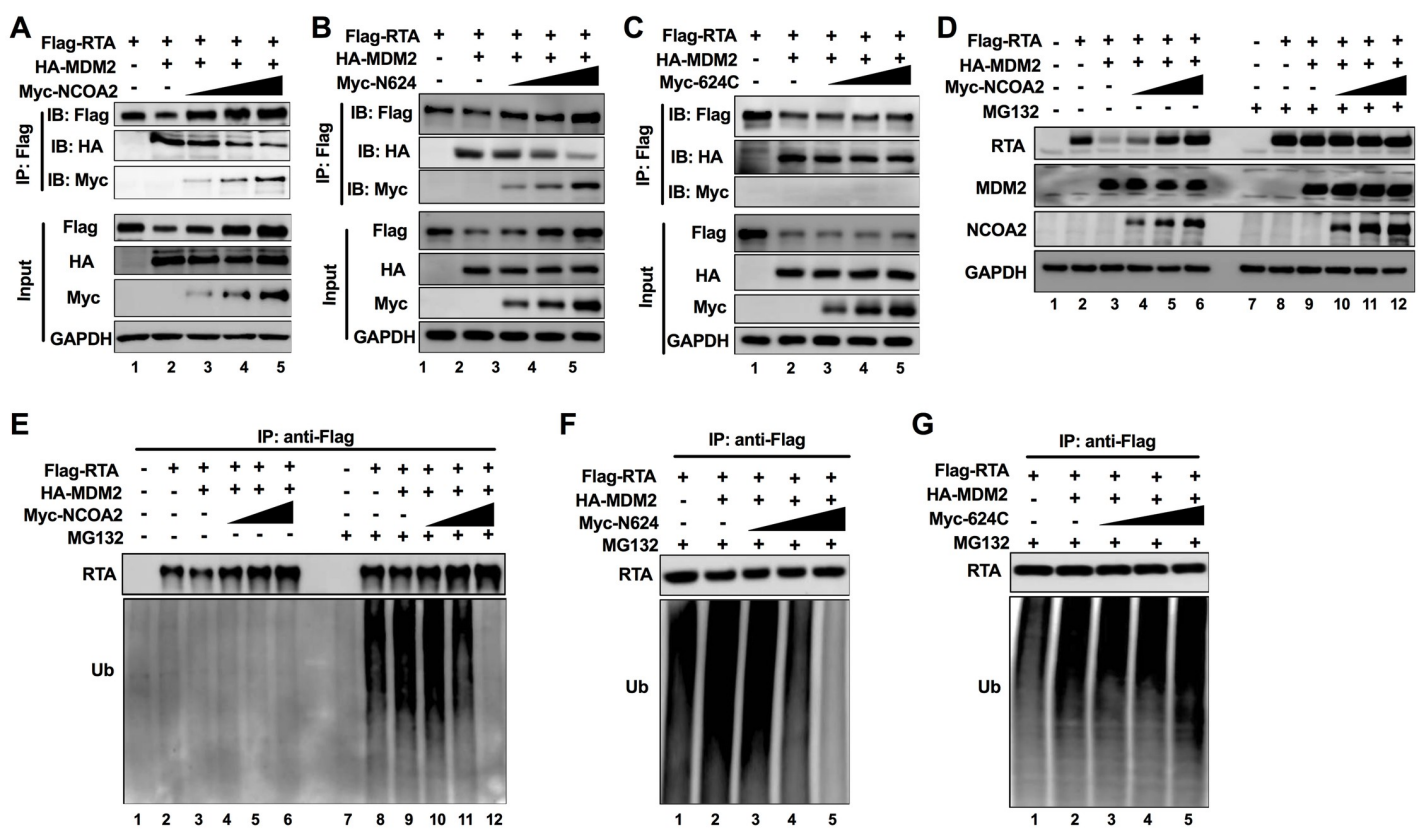
NCOA2 inhibits the ubiquitination of RTA by blocking the interaction of RTA with MDM2. (A-C) NCOA2 disrupts the interaction between RTA and MDM2. 293T cells were transiently transfected with Flag-RTA and HA-MDM2 together with an increasing amount of Myc-NCOA2 (A), Myc-N624 (B) or Myc-624C (C) (0, 0.5, 1, 2 μg). Forty-eight hours after transfection, the cell lysates were collected and subjected to immunoprecipitation using an anti-Flag antibody. Purified proteins, along with input samples, were detected by western blotting with the indicated antibodies. (D) NCOA2 inhibits the proteasome-mediated degradation of RTA induced by MDM2. 293T cells were transiently transfected with Flag-RTA and HA-MDM2 together with an increasing amount of Myc-NCOA2 (0, 0.5, 1, 2 μg) for 36 h, and then, they were treated with 0.5 μM MG132 or 0.1% DMSO for another 6 h. The cells were lysed and used for western blots with the indicated antibodies. (E) NCOA2 inhibits the ability of MDM2 to increase the ubiquitination of RTA. The same lysates as in (D) were subjected to immunoprecipitation using anti-Flag antibody, followed by western blotting with anti-ubiquitin antibodies. (F-G) 293T cells were transiently transfected with Flag-RTA and HA-MDM2 together with an increasing amount of Myc-N624 (F) or Myc-624C (G) (0, 0.5, 1, 2 μg) for 36 h, then cells were treated with 0.5 μM MG132 for another 6 h. Cells were lysed and subjected to immunoprecipitation using anti-Flag antibody, followed by western blotting with anti-ubiquitin antibodies.

As the C-terminal PARS II domain of RTA serves as the binding site for E3 ubiquitin enzymes to modulate the degradation of RTA [35], the interaction between the PARS II domain of RTA and NCOA2 is likely to impair the interaction between RTA and host E3 ubiquitin ligases. Previously, the host protein MDM2 was reported to be the E3 ubiquitin ligase of RTA, which can mediate the degradation of RTA [35]. Interestingly, MDM2 was also shown to bind the PARS domain of RTA. We therefore hypothesized that NCOA2 may block MDM2 binding to the PARS domain of RTA by interacting with the PARS II domain of RTA and consequently preventing RTA ubiquitination and enhancing the stability of RTA. To test this hypothesis, 293T cells were cotransfected with RTA, MDM2 and NCOA2, and the binding of RTA to MDM2 or NCOA2 was examined by Co-IP. As previously reported, MDM2 reduced the steady-state level of RTA [35] (Fig 6A, lane 2). Moreover, we found that MDM2-mediated reduction in RTA expression was inhibited by NCOA2 in a dose-dependent manner (Fig 6A, lanes 3–5). As NCOA2 increased, the formation of the RTA-MDM2 complex was largely impaired; however, the RTA-NCOA2 complex was increased (Fig 6A). The mutant N624, which interacts with RTA, has same effect (Fig 6B), while 624C that cannot bind to RTA abolished the ability to compete with MDM2 for binding to RTA (Fig 6C). Thus, NCOA2 affected RTA expression level by blocking the interaction between RTA and MDM2.

MDM2 functions as a ubiquitin E3 ligase of RTA that mediates the degradation of RTA through the ubiquitin-proteasome pathway. As shown above, NCOA2 expression impaired MDM2-mediated degradation of RTA (Fig 6A). We then tested whether NCOA2 inhibits the proteasome-associated degradation of RTA caused by MDM2. The 293T cells were cotransfected with RTA, MDM2 and NCOA2 and then treated with MG132. In the absence of MG132, NCOA2 inhibited the ability of MDM2 to degrade RTA protein (Fig 6D, lanes 1–6); however, in the presence of the proteasome inhibitor MG132, the abundance of RTA was dramatically enhanced and did not change as NCOA2 increased (Fig 6D, lanes 7–12). For further analysis of the effect of NCOA2 on the ubiquitination of RTA modulated by MDM2, the cell extracts were immunoprecipitated with anti-Flag antibody that pulled down Flag-tagged RTA, followed by western blotting with an anti-ubiquitin antibody. In cells that expressed MDM2 and were treated MG132, we observed an increase in the ubiquitin level of RTA compared with that in cells lacking MDM2 expression (Fig 6E, lanes 8 and 9). As expected, NCOA2 inhibited the ability of MDM2 to increase the ubiquitination of RTA (Fig 6E, lanes 10–12). Additionally, overexpression of N624 also inhibited the ability of MDM2 to increase the ubiquitination of RTA in a dose-dependent manner (Fig 6F). However, 624C had no effect on the ubiquitination level of RTA that is regulated by MDM2 (Fig 6G).

Taken together, these results suggested that NCOA2 targets RTA and competes with MDM2 for RTA binding. Therefore, the association of the MDM2-RTA complex can be disrupted by NCOA2, resulting in a reduction in ubiquitination of RTA by MDM2 and an increase in the stability of RTA protein.

### Ectopic expression of NCOA2 enhances KSHV lytic reactivation

**Fig 7:**
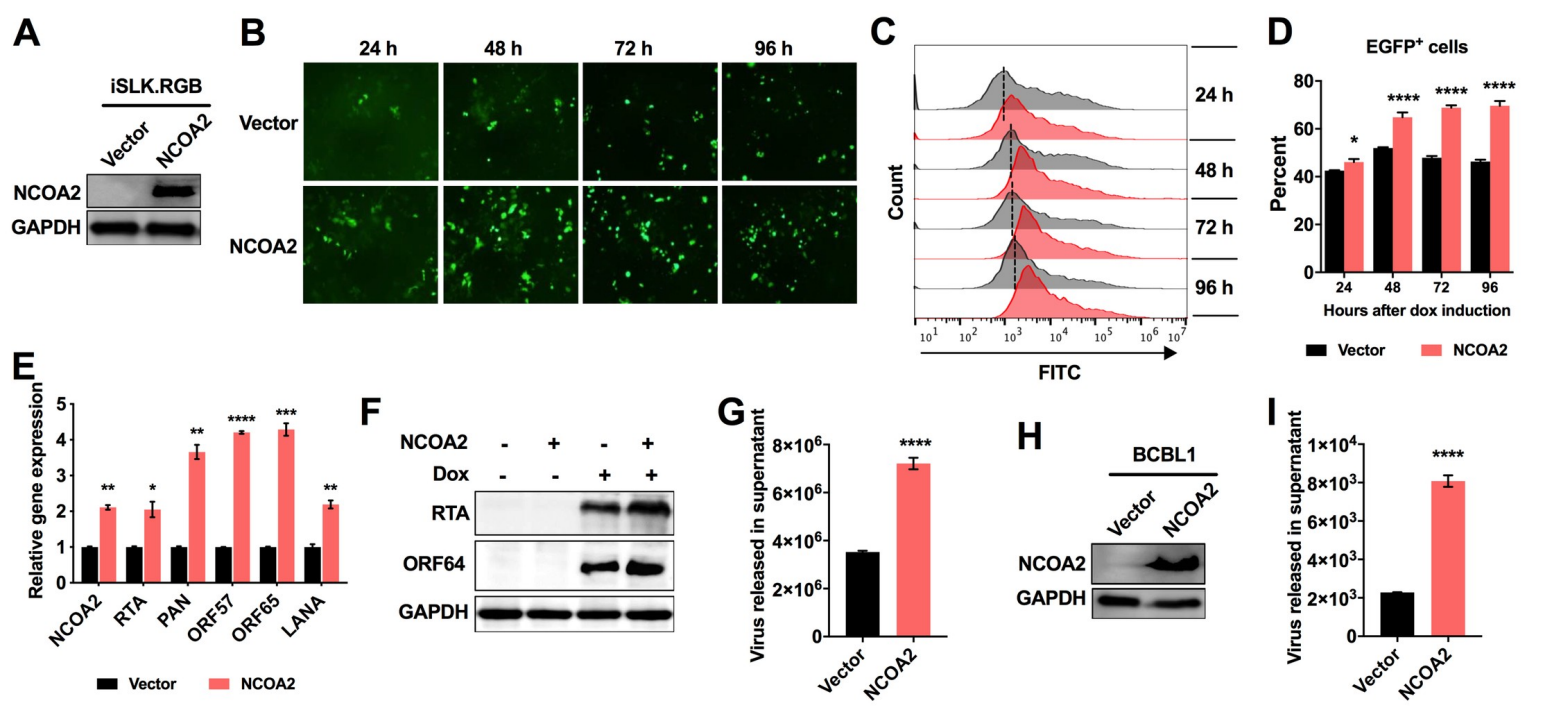
Ectopic expression of NCOA2 enhances KSHV lytic reactivation. (A) iSLK.RGB cells were stably transfected with lentiviruses containing a Flag-tagged NCOA2 expression plasmid or an empty vector plasmid, named iSLK.RGB-NCOA2 and iSLK.RGB-Vector, respectively. The overexpression of NCOA2 was detected by western blotting. (B) iSLK.RGB-Vector and iSLK.RGB-NCOA2 cells were treated with dox at different time points as indicated. Fluorescence microscopy images of EGFP-positive cells among iSLK.RGB-Vector and iSLK.RGB-NCOA2 cells. (C) Flow cytometry analysis of EGFP-positive cells among iSLK.RGB-Vector and iSLK.RGB-NCOA2 cells. (D) Quantitation of the percent of EGFP-positive cells from (C). (E) NCOA2 overexpression increases the transcription of viral genes. RNA was extracted from dox-induced cells at 48 hours post-induction (hpi) to investigate the transcriptional level of NCOA2 and several KSHV genes: RTA, PAN, ORF57, ORF65 and LANA. (F) NCOA2 overexpression increases the expression of viral genes. The expression levels of RTA protein and ORF64 protein were determined by immunoblotting with the indicated antibodies. (G) NCOA2 overexpression increases virus production. Culture supernatants from dox-induced iSLK.RGB-Vector and iSLK.RGB-NCOA2 cells at 48 hpi were quantified by qPCR for KSHV copy numbers. (H) BCBL1 cells were stably transfected with lentiviruses containing a NCOA2 expression plasmid or an empty vector plasmid, named BCBL1-NCOA2 and BCBL1-Vector, respectively. The overexpression of NCOA2 was detected by western blotting. (I) NCOA2 overexpression increases virus production in BCBL1 cells. BCBL1-NCOA2 and BCBL1-Vector cells were treated with VPA for 24 h, and the KSHV copy numbers from culture supernatants were quantified by qPCR. Data in D, E, G, and I represent the mean +/- SD of 3 replicates pooled from three independent experiments. Data were analyzed with a two-tailed Student’s *t*-test (*P < 0.05; **P < 0.01; ***P < 0.001; ****P < 0.0001).

NCOA2 enhanced the stability of RTA, suggesting that NCOA2 functions as a positive modulator of RTA. As a result, this protein may promote viral lytic replication. To explore the effect of NCOA2 on RTA expression in the context of KSHV-infected cells, we used the KSHV-positive iSLK.RGB cell line that was stably infected with a reporter KSHV virus called red-green-blue-BAC16, which harbored three fluorescent protein expression cassettes: EF1α-monomeric red fluorescent protein 1, polyadenylated nuclear RNA promoter (pPAN)-enhanced green fluorescent protein (EGFP), and pK8.1-monomeric blue fluorescent protein, marking latent, immediate early and late viral gene expression, respectively. Therefore, KSHV-infected cells were indicated by red fluorescence, and cells with lytic KSHV infection were indicated by green fluorescence [41]. iSLK cells stably express dox-inducible RTA [42]. The expression of RTA was essential and sufficient for triggering KSHV lytic replication. iSLK.RGB cells were used to construct two stable transfected cell lines by transduction with lentivirus vector expressing a Flag-tagged NCOA2 and an empty vector as a control, yielding iSLK.RGB-NCOA2 and iSLK.RGB-Vector, respectively (Fig 7A). Lytic replication of KSHV in these two cell lines was induced by dox-induced RTA expression. We examined the ratio of cells with lytic KSHV infection by fluorescence microscopy, as the lytic KSHV-infected cells expressed EGFP. The expression of EGFP was increased in NCOA2-overexpressing cells compared with control cells (Fig 7B). Similar results were also observed when the cells were quantified by flow cytometry, and the number of EGFP-positive cells was increased in the iSLK.RGB-NCOA2 stable cell lines (Fig 7C and 7D). After dox-induced RTA expression for 48 h, we examined the transcript levels of various viral genes by real-time qPCR. As shown in Fig 7E, the transcription levels of both the lytic and latent genes of KSHV were increased in the NCOA2 stable expression cell lines. The expression levels of KSHV lytic genes were further confirmed by western blotting. As expected, NCOA2 overexpression increased the expression levels of RTA and the late lytic gene ORF64 (Fig 7F). Furthermore, the KSHV virions released into the supernatant of these two cell cultures were detected. We found that NCOA2 overexpression resulted in a significant increase in the production of KSHV virions compared to that in the iSLK.RGB-Vector cell line (Fig 7G). The progeny viruses prepared from cell culture of iSLK.RGB-NCOA2 and iSLK. RGB-Vector cells were also used to infect 293T cells in the same volume of cell culture, and increased red fluorescence was observed in the NCOA2-overexpressing group (S4A Fig). To strengthen these results, we used another KSHV-positive cell line, BCBL1, to establish the BCBL1-NCOA2 cell line and BCBL1-Vector cell line (Fig 7H) and then treated the cells with VPA. Similar results were obtained from BCBL1 cells. The transcription of viral genes, the expression of viral genes and virion production were substantially increased in the NCOA2-overexpressing BCBL1 cell lines (Figs 7I and S4B and S4C). Taken together, these results suggested that NCOA2 can promote KSHV lytic replication by enhancing the expression of RTA.

### Downregulation of endogenous NCOA2 impairs viral lytic replication

**Fig 8:**
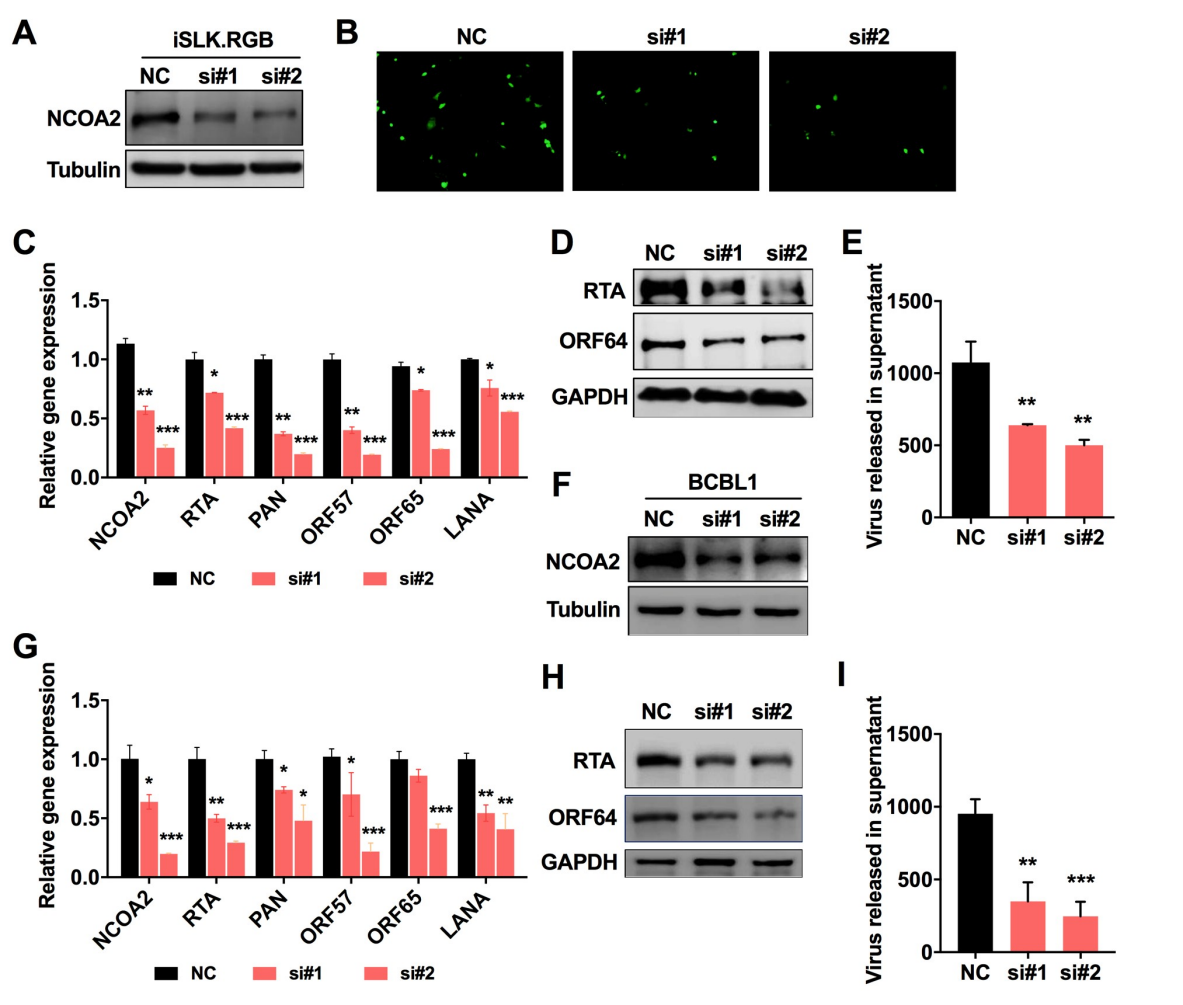
Knockdown of endogenous NCOA2 impairs KSHV lytic replication. (A) iSLK.RGB cells were transfected with control siRNA and two NCOA2-specific siRNAs. The knockdown efficiency was determined by western blotting. At 24 h after transfection, cells were induced by dox for another 24 h. (B) EGFP-positive cells were analyzed by fluorescence microscopy. (C) The KSHV gene transcription level was analyzed by qPCR. (D) The expression levels of RTA and ORF64 were examined by western blotting. (E) The progeny viruses from culture supernatants were analyzed by qPCR. (F) BCBL1 cells were also transfected with control siRNA and two NCOA2-specific siRNAs. The knockdown efficiency was determined by western blotting. At 24 h after transfection, cells were induced by VPA for another 24 h. The KSHV gene transcription level (G), the expression levels of RTA and ORF64 (H) and the progeny viruses (I) were analyzed by the same approaches that were used in iSLK.RGB stable cell lines. Data in C, E, G, and I represent the mean +/- SD of 3 replicates pooled from three independent experiments. Data were analyzed with a two-tailed Student’s *t*-test (*P < 0.05; **P < 0.01; ***P < 0.001).

To further confirm the effect of NCOA2 on KSHV lytic replication, we transfected iSLK.RGB cells with two NCOA2-specific siRNAs or equal amounts of control siRNA 24 h before induction with dox. The siRNAs reduced the NCOA2 protein levels by 68% and 78% (Fig 8A). Knockdown of NCOA2 in iSLK.RGB cells decreased the expression of EGFP (Fig 8B), the transcription of viral genes (Fig 8C), the expression levels of viral RTA and ORF64 protein (Fig 8D), and virion production (Fig 8E) compared to those in cells transfected with the scrambled siRNA control. Similar results were observed in NCOA2-specific siRNA-transfected BCBL1 cells (Fig 8F), in which KSHV lytic replication was induced by VPA (Fig 8G–8I). To further confirm these results, we generated NCOA2-deficient iSLK.RGB cell line. The deletion of NCOA2 was confirmed by western blotting (S5A Fig). The results showed that the expression levels of viral proteins (S5A Fig) and virions (S5B Fig) in NCOA2-deficient cells were dramatically reduced compared to wild-type iSLK.RGB cells. Collectively, these results indicate that NCOA2 promotes the lytic replication of KSHV through enhancing RTA expression.

### Expression of NCOA2 is upregulated during viral lytic replication by RTA

**Fig 9:**
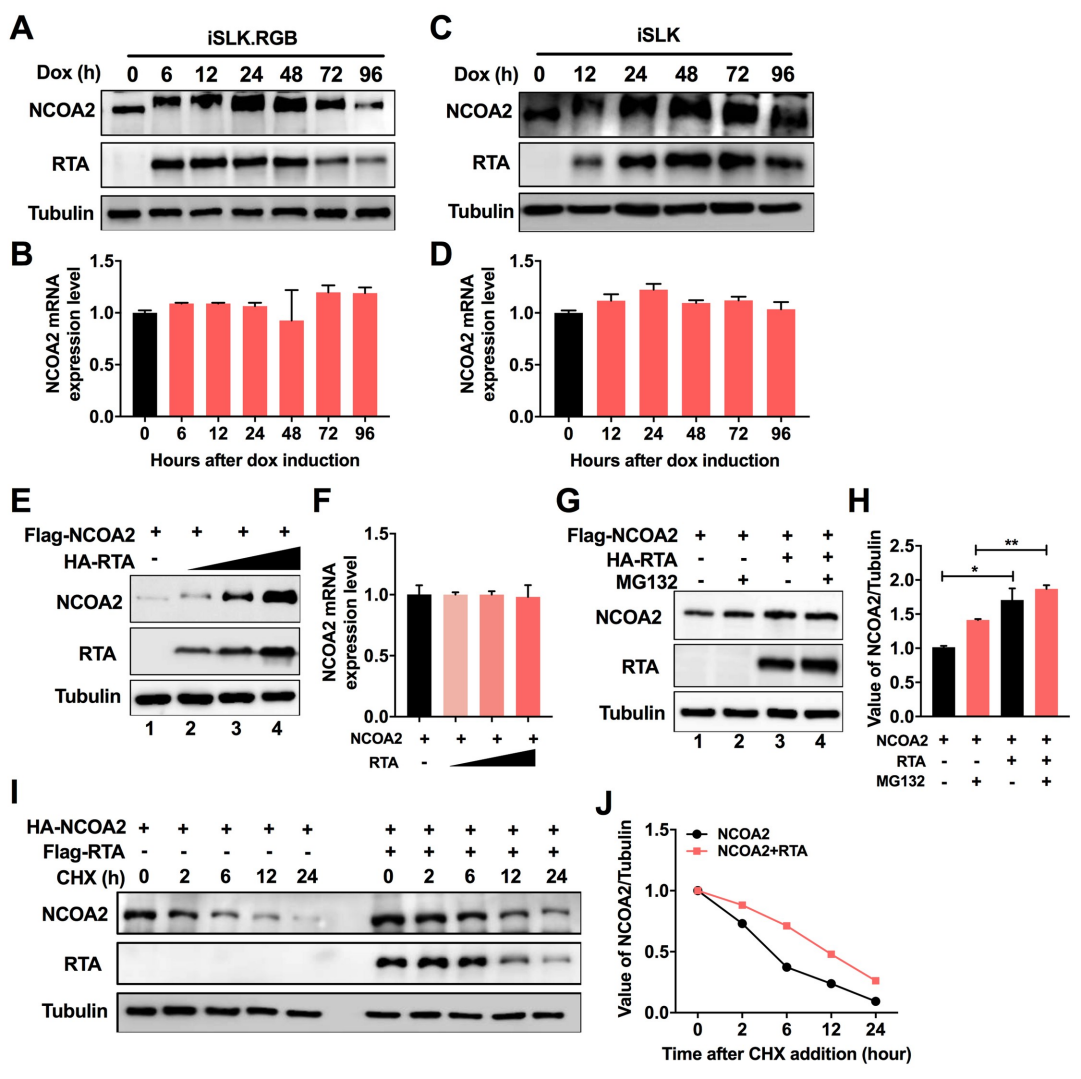
Expression of NCOA2 is upregulated during viral lytic replication by RTA. (A and C) The expression kinetics of NCOA2 and RTA were analyzed in iSLK.RGB cells (A) or iSLK cells (C) at indicated time points after dox treatment. (B and D) NCOA2 mRNA expression level in cells from (A) or (C). (E) Effect of RTA on NCOA2 expression. 293T cells were cotransfected with 1 μg of NCOA2 expression plasmid and increasing amounts of RTA expression vector (0, 0.5, 1, 2 μg). The protein level of NCOA2 was examined by immunoblotting. (F) NCOA2 mRNA expression level in cells from (E). (G) RTA inhibits the degradation of NCOA2. 293T cells were cotransfected with the indicated expression constructs for 36 h and then treated with 0.5 μM MG132 or 0.1% DMSO for another 6 h. Cells were lysed and used for western blots with the indicated antibodies. The band intensities of NCOA2 were plotted graphically (H). Data were from a minimum of three experimental replicates with a standard deviation. Data were analyzed by unpaired *t*-test. *, *P* < 0.05; **, *P* < 0.01. (I) Measurement of NCOA2 stability in the absence and presence of RTA. 293T cells were transfected with HA-tagged NCOA2 with or without Flag-tagged RTA expression plasmid for 36 h. Cells were treated with 100 μg/ml of CHX and analyzed at indicated time points by immunoblotting for NCOA2. Tubulin was used as a control for equivalent sample loading. (J) The relative levels of NCOA2 from (I) were quantified by densitometry and normalized to the Tubulin level.

The above results demonstrated that NCOA2 functions as a positive modulator of RTA, which facilitates viral lytic replication. We examined the kinetics of NCOA2 expression in iSLK.RGB cells during the induction of viral lytic reactivation. Interestingly, upon treatment with dox, RTA protein expression was induced, and the NCOA2 level was also increased along with the increasing expression of RTA (Fig 9A). These results suggest that a positive feedback loop between RTA and NCOA2 may exist during viral lytic reactivation. However, no significant changes were observed in NCOA2 mRNA levels during the induction of viral lytic reactivation (Fig 9B). To further determine whether NCOA2 expression is responsive to RTA, we examined the kinetics of NCOA2 expression in iSLK cells that stably express dox-inducible RTA. Upon the induction of RTA protein by dox, we observed an increase in NCOA2 expression and a subsequent decrease following the reduced expression of RTA at 72 h post-induction (Fig 9C). The mRNA level of NCOA2 was also unchanged (Fig 9D). An increasing amount of RTA was coexpressed with a constant amount of NCOA2 in 293T cells, and the effect of RTA on NCOA2 expression was examined. We observed that NCOA2 expression was enhanced by RTA in a dose-dependent manner (Fig 9E), whereas there were no significant changes in the mRNA levels of NCOA2 between cells with or without RTA expression (Fig 9F). As shown above, NCOA2 was subjected to ubiquitin-proteasome mediated degradation (Fig 5). To address the mechanism by which NCOA2 is regulated by RTA, 293T cells that cotransfected with NCOA2 and RTA were treated with MG132. In the absence of RTA, the steady-state of NCOA2 was increased moderately in cells exposed to MG132 (Fig 9G and 9H, lanes 1 and 2). However, in the presence of RTA, the abundance of NCOA2 was enhanced significantly regardless of the presence or absence of MG132 (Fig 9G and 9H, lanes 3 and 4), indicating that there may exist other mechanisms to govern RTA-mediated NCOA2 turnover beyond the proteasome. We also examined the half-life of NCOA2 by treating cells with CHX in 293T cells. We found that both the steady-state level and half-life of NCOA2 were increased in cells overexpressing RTA as compared with the control group (Fig 9I and 9J). These results indicated that RTA also inhibits the degradation of NCOA2 and demonstrated a positive regulatory loop between RTA and NCOA2.

## Discussion

**Fig 10:**
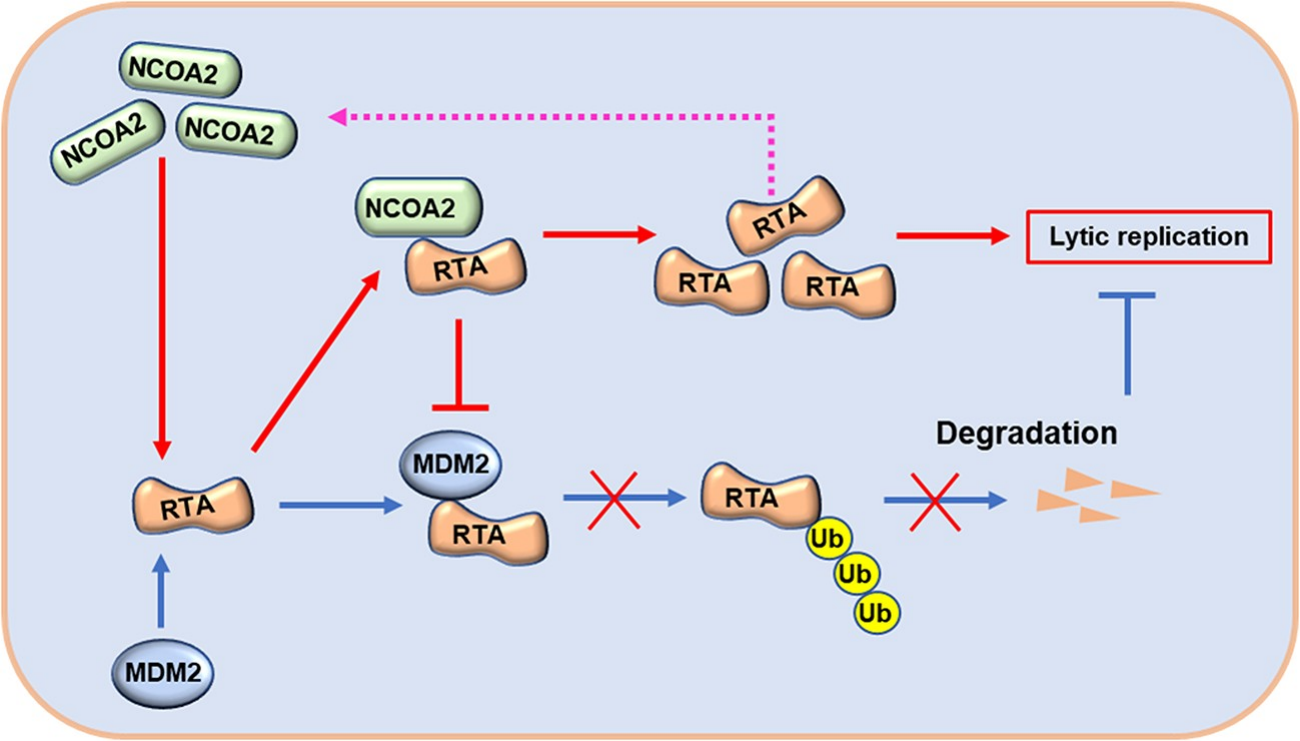
Working model for the role of NCOA2 in regulating the stability of RTA. After interacting with RTA, MDM2 promotes the ubiquitination and degradation of RTA, which inhibits KSHV lytic replication. NCOA2 competes with MDM2 to interact with RTA, which blocks the interaction between RTA and MDM2 and then inhibits the degradation of RTA. The abundance of RTA is increased by NCOA2. In turn, RTA promotes the expression of NCOA2, which forms a positive feedback loop between NCOA2 and RTA and promotes KSHV lytic replication.

In this study, we found that NCOA2 interacts with the PARS II domain of RTA, which prevents the degradation of RTA by MDM2, an E3 ligase, also bound to the same domain of RTA, promoting RTA-triggered KSHV lytic reactivation. During lytic reactivation, NCOA2 stabilizes RTA to increase the RTA level in the cells, ensuring that KSHV can be switched from latency to the lytic phase. In addition, NCOA2 was elevated in response to RTA expression during KSHV lytic reactivation. This finding suggests that there is a positive feedback regulation loop between NCOA2 and RTA, which facilitates a complete lytic reactivation program driven by RTA. In summary, our data establish a working model illustrating the role of NCOA2 in the regulation of RTA and KSHV lytic reactivation (Fig 10).

In all KSHV-related malignancies, the resulting tumor cells predominantly display latent viral infection. Latency alone, however, is not sufficient to sustain tumorigenesis *in vivo*. Clinical trials and many studies indicate an important role for lytic infection in tumor development [43–47]. The lytic cycle is essential for producing progeny virus to disseminate and infect new cells to replace those that have segregated the viral episome [11, 13]. Therefore, both latency and lytic replication are important for viral oncogenesis. RTA functions as a lytic switch protein that is essential and sufficient for the lytic reactivation of KSHV from latency. Overexpression of RTA disrupted viral latency and induced the virus to undergo the complete lytic replication process [20, 48]. This result is due to its ability to transcriptionally activate numerous viral genes and assemble replication complexes that are required for lytic DNA synthesis. RTA realizes its multiple functions through interactions with many different viral or host proteins, such as Oct-1, RBP-Jκ, and C/EBPα as transcription cofactors to enhance the transcriptional activity of RTA, and in turn increases viral gene expression [22, 26, 49]. In contrast, PARP-1/hKFC, K-RBP and TLE2, which were identified as transcriptional repressors, interact with RTA to impair RTA-mediated viral gene expression [30, 50, 51]. As a multifunctional protein, RTA abundance must be subjected to exquisite control for optimal KSHV lytic-cycle gene expression and viral DNA synthesis. Therefore, understanding the molecular mechanisms involved in regulating the expression of RTA not only contributes to understanding the regulation of the viral reactivation process but also elucidates the pathogenesis mediated by the virus, which may contribute to the development of potential effective therapeutics. In the present study, we found that KSHV utilizes NCOA2 to enhance the stability of RTA to increase the abundance of RTA, thereby promoting viral lytic replication. The results suggest that targeting NCOA2 might be a strategy for lytic phase-based therapeutic approaches for controlling KSHV infection and related diseases.

Ubiquitin-mediated proteolysis plays an important role in controlling viral infection through degradation of key viral proteins. Pey-Jium Chang et al. demonstrated that the abundance of RTA is regulated by the ubiquitin-proteasome degradation system and is controlled through a PARS domain that contains PARS I and PARS II within its C-terminal region [36]. A previous study demonstrated that the regulation of the RTA degradation process is divided into two successive steps: i) the nuclear translocation of RTA, which is controlled by the PARS I domain, and ii) the PARS II domain binding the host E3 ubiquitin ligase to cause the degradation of RTA in the nucleus [35]. MDM2 is a host E3 ubiquitin ligase that interacts with RTA and causes the ubiquitin degradation of RTA in the nucleus. A predicted MDM2-binding motif is present within the PARS II domain (592 aa–610 aa); in fact, the whole PARS domain is necessary for binding to MDM2 [35]. Interestingly, another group found that the transcript from the opposite strand of the RTA DNA template encodes a small peptide, namely, vSP1, which has been proven to interact with RTA in the nucleus. Further studies showed that vSP1 increases RTA stability by binding to the PARS II domain of RTA and inhibits the proteasome-mediated degradation of RTA [37]. As both vSP1 and MDM2 bind to the PARS domain, the expression of vSP1 may facilitate the dissociation of RTA from MDM2. In our study, we identified the first host factor, NCOA2, which disrupts the association between MDM2 and RTA through completely binding to the PARS II domain of RTA. Like vSP1, which was utilized by KSHV, NCOA2 also stabilizes the RTA protein in viral lytic reactivation. Although we only observed a slight increase in RTA expression when co-transfected with NCOA2 and vSP1 compared to NCOA2 alone or vSP1 alone, the relationship between vSP1 and NCOA2 in controlling RTA levels remains to be further studied. This finding suggests that KSHV uses either the small peptide encoded by itself or the host factor to prevent the degradation of RTA during the viral reactivation process so that the virus can ensure a sufficient level to drive the complete lytic program. However, unlike vSP1, which is a transcription product of the KSHV lytic phase and is detected at 24 h and 48 h post-induction, we found that NCOA2 is expressed in different cell types even before lytic reactivation of KSHV. To ensure that the threshold level of RTA expression and the completion of the lytic cascade are achieved, KSHV has developed an efficient strategy to hijack the host protein NCOA2 against RTA degradation.

NCOA2, also known as glucocorticoid receptor-interacting protein 1 (GRIP1), steroid receptor coactivator-2 (SRC-2) or transcriptional mediators/intermediary factor 2 (TIF2), is a member of the p160 family of transcriptional coactivators [52]. NCOA2 cooperates with NRs controlling multiple physiological processes, including glucose homeostasis, energy metabolism and reproduction [53, 54]. Interestingly, NCOA2 is widely known for its oncogenic role, and NCOA2 gene fusion, mutations, deletions, insertions and overexpression have been observed in multiple cancers, including endometrial cancer, pleural cancer and breast cancer [55–57]. However, the biological roles of NCOA2 in virus infection remain elusive. In this study, we discovered a novel mode of action of NCOA2 that interacts with RTA to increase its stability and protein abundance, ultimately promoting the lytic replication of KSHV. NCOA2 also serves as a binding platform for additional cofactors, such as CBP/p300, CARM1 and PRMTs, to regulate transcription factor activation [58]. Therefore, RTA may utilize NCOA2 as an adaptor molecule to recruit other host factors to enhance the function of RTA, which requires further study.

Deng et al. previously reported that RTA promotes transcription by activating its promoter [21]. The autoactivation of RTA is an important strategy for enhancing the transcription level of RTA and then activating virus lytic replication. NCOA2 functions as a positive regulator that increases the protein level of RTA and then promotes virus lytic replication. Our findings extend the understanding of the molecular mechanism by which RTA abundance is modulated. Interestingly, we also found that the protein level of NCOA2 elevated during viral lytic reactivation process, and the kinetics of NCOA2 were highly correlated with RTA expression. These results support the existence of a positive feedback loop between NCOA2 and RTA during viral reactivation process. Although our preliminary data suggest that RTA inhibits the proteasome-mediated degradation of NCOA2, which may be realized through blocking the host E3 ubiquitin ligase from binding to NCOA2, we cannot exclude the possibility that other mechanisms to govern RTA-mediated NCOA2 turnover beyond the proteasome. The detailed mechanism by which NCOA2 is upregulated requires further investigation.

In summary, our work identified for the first time that NCOA2 is a novel RTA-binding protein and that NCOA2 functions as a positive modulator of RTA-driven lytic reactivation. Due to the important function of NCOA2 in the KSHV lytic reactivation process, inhibition of NCOA2 expression in KSHV-infected cells would be a potentially effective therapeutic approach for KSHV infection and related diseases.

## Materials and methods

### Cells and reagents

The iSLK.RGB cells, which harbor wild-type BAC16.RGB, were kind gifts from Jae Jung (University of South California, Los Angeles, USA) and were cultured in Dulbecco’s modified Eagle’s medium (DMEM, HyClone) supplemented with 10% FBS (Biological Industries) and 1% antibiotics (penicillin and streptomycin, Gibco). KSHV-positive B lymphoma cell lines (BCBL1, JSC1, and BC3) were cultured in RPMI 1640 (HyClone) supplemented with 10% FBS and 1% antibiotics. Human embryonic kidney (HEK) 293T and HeLa cell lines were cultured in DMEM supplemented with 10% FBS and 1% antibiotics. The stable cell lines iSLK.RGB-NCOA2, iSLK.RGB-Vector, BCBL1-NCOA2 and BCBL1-Vector were established by infection with the indicated lentiviruses according to the manufacturer’s instructions (System Bioscience, Palo Alto, USA). All cell lines were grown at 37°C in a humidified environment supplemented with 5% CO_2_.

Dox, VPA, CHX, MG132 and DNase I were purchased from Sigma-Aldrich (St. Louis, MO), as was an anti-Flag M2 affinity gel (Sigma, A2220).

### Plasmids and antibodies

The NCOA2 construct was amplified from a BCBL1 cell cDNA library, inserted into pCMV-HA at EcoRI and KpnI sites, and subcloned into the pCDH-Flag (pCDH) vector at XhoI and BamHI sites and pGEX-4T at BamHI and XhoI sites. pCDH-Flag-RTA, which encodes the full-length RTA, has been described previously [59]. RTA was further subcloned into the expression vectors pCMV-HA and pET-30a. pCDH expression plasmids encoding truncated NCOA2 fragments, including N765, 765C, N1007, N624, and 624C, which are schematically shown in Fig 3A, were obtained by PCR amplification of pCDH-Flag-NCOA2. Truncated forms of RTA with Flag tags, including N273, N544, RTA273-544, C544-691, C273-691, and RTA-△PARS-II, which are schematically shown in Fig 3C and 3F, were amplified from the full-length template and cloned into the pCDH vectors. All constructs were verified by DNA sequencing (Sangon Biotech Co., Ltd., Shanghai, China). The MDM2 gene was amplified from a 293T cell cDNA library and inserted into pCMV-HA at the EcoRI and KpnI sites. Flag-vSP1 was a gift from Dr. Yan Yuan’s lab (University of Pennsylvania, Philadelphia, Pennsylvania USA). sh-NCOA2 was cloned into pLKO.1, and the target sequence of NCOA2 shRNA is 5'- ATCCGTTCTCAGACTACTAAT-3'. A control shRNA (sh-GFP) was also cloned into pLKO.1 (target sequence: 5'-GCTACCTGTTCCATGGCCAA-3'). The PCR primers used in this study are summarized in Table 1.

**Table 1:** Primers used in this study.

Primer name	Purpose	Sequence of Oligonucleotide (5′-3′)	
**pCMV-HA**			
NCOA2	Full length	F CGGAATTCGGATGAGTGGGATGGGAGA	
	Cloning	R GGGGTACCTCAGCAATATTTCCGTGT	
RTA	Full length	F CGGAATTCGGATGGCGCAAGATGACAAGGGT	
	Cloning	R GAAGATCTTCAGTCTCGGAAGTAATTACGCC	
MDM2	Full length	F CGGAATTCGGGCCACCATGGTGAGGAGCAGG	
	Cloning	R GGGGTACCGGGGAAATAAGTTAGCACAATC	
**pCDH-Flag**			
NCOA2	Full length	F CCGCTCGAGATGAGTGGGATGGGAGAAAATAC	
	Cloning	R CGGGATCCGCAATATTTCCGTGTTGTGTCT	
N765	Truncated	F CGCTCGAGATGAGTGGGATGGGAGAAAATAC	
	Cloning	R CGGGATCCTTTGGGGGTTATTTCTGGT	
765C	Truncated	F CCGCTCGAGGCCACCAAACTTGAGAGACTGG	
	Cloning	R CGGGATCCGCAATATTTCCGTGTTGTGTCT	
N1007	Truncated	F CCGCTCGAGATGAGTGGGATGGGAGAAAATAC	
	Cloning	R CGGGATCCCATGACCTGAGACTGAAGCGT	
N624	Truncated	F CCGCTCGAGATGAGTGGGATGGGAGAAAATAC	
	Cloning	R CGGGATCCTTTGCTGTCATGCAGTCTG	
624C	Truncated	F CCGCTCGAGATGAAAGGGCAGACCAAACT	
	Cloning	R CGGGATCCGCAATATTTCCGTGTTGTGTCT	
RTA	Full length	F CCGCTCGAGATGGCGCAAGATGACAAG	
	Cloning	R CGGAATTCTCGTCTCGGAAGTAATTACGC	
N273	Truncated	F CCGCTCGAGATGGCGCAAGATGACAAG	
	Cloning	R CGGAATTCTCCATGGAAGCCGGCAACAGT	
N544	Truncated	F CCGCTCGAGATGGCGCAAGATGACAAG	
	Cloning	R CGGAATTCTCCGTGGTCGATGGCGTGGT	
RTA273-544	Truncated	F CCGCTCGAGATGGTAGACCTCAGCGATG	
	Cloning	R CGGAATTCTCCGTGGTCGATGGCGTGGT	
C544-691	Truncated	F CCGCTCGAGATGACGACACCTGGTACCTC	
	Cloning	R CGGAATTCTCGTCTCGGAAGTAATTACGC	
C273-691	Truncated	F CCGCTCGAGATGGTAGACCTCAGCGATG	
	Cloning	R CGGAATTCTCGTCTCGGAAGTAATTACGC	
RTA-△-PARS-II	Deletion	F CCTTCTGGTGGAGAGTATACGCAAC	
	Cloning	R ACTCTGATCTACGTCCAGTGGCGGG	
Myc-NCOA2	PCR	F CGGGATCCATGAGTGGGATGGGAGAAAATACCT	
	Cloning	R CCATCGATGCAATATTTCCGTGTTGTGTCT	
GST-NCOA2	PCR	F CGGGATCCATGAGTGGGATGGGAGAAAATACCT	
	Cloning	R CCGCTCGAGGCAATATTTCCGTGTTGTGTCT	
His-RTA	PCR	F CGGAATTCATGGCGCAAGATGACAAG	
	Cloning	R CCGCTCGAGGTCTCGGAAGTAATTACGC	
His-C544-691	PCR	F CGGAATTCATGACGACACCTGGTACCTC	
	Cloning	R CCGCTCGAGCGTGGTCGATGGCGTGGT	
NCOA2	qRT-PCR	F AACAAATGACCCCAACCTGC	
		R TAGACCCAGAACCAGGCAAG	
RTA	qRT-PCR	F CGTGTAGAGATTCAACGGCG	
		R AAGAGGTACCAGGTGTCGTG	
PAN	qRT-PCR	F GCCGCTTCTGGTTTTCATTG	
		R TTGCCAAAAGCGACGCA	
ORF57	qRT-PCR	F GAGGTGTTTACGGACAGGGA	
		R CCCACGTCATTTGTTCCTCC	
ORF65	qRT-PCR	F ATGACTACGCTCACCATCCC	
		R CGCCTTTGAATTCCACCCAT	
LANA	qRT-PCR	F GCAGACTACACCTCCACACT	
		R GTAGATCGGGGACTCTGTGG	
K9	qRT-PCR	F GTCTCTGCGCCATTCAAAAC	
		R CCGGACACGACAACTAAGAA	
GAPDH	qRT-PCR	F AAATTGTCAGCAATGCCTCTTG	
		R GGCATGGACAGTGGTCATAA	

The following primary antibodies were used: anti-NCOA2 rabbit monoclonal antibody (CST, #96687), anti-RTA (prepared in our laboratory), anti-ORF64 (a kind gift from Yan Yuan, University of Pennsylvania, Philadelphia, Pennsylvania USA), anti-ubiquitin antibody (ABclonal, A3207), anti-GST (ABclonal, AE001) anti-His (ABclonal, AE003), anti-Flag antibody (Sigma, F1804), anti-HA (Sigma-Aldrich, H6908), anti-GAPDH (Sigma-Aldrich, G8795), and anti-α-Tubulin antibody (Sigma-T6199). The secondary antibodies used in western blotting and immunofluorescence assays were HRP-conjugated anti-mouse or anti-rabbit IgG (Jackson ImmunoResearch Laboratories) and goat anti-mouse antibodies conjugated with Alexa Fluor 488 (Thermo Fisher Scientific, A-11029) and 555 (Thermo Fisher Scientific, A-21422).

### Mass spectrometry

Flag-tagged RTA or the empty vector was overexpressed in HEK293T and HEK293T.219 cell lines. After 48 h, the cells were washed two or three times with pre-cooled PBS and lysed in ice-cold radioimmunoprecipitation assay (RIPA) buffer [50 mM Tris pH 7.6, 150 mM NaCl, 2mM EDTA, 0.5 Nonidet P-40, 1 mM protease inhibitor (PMSF) and protease inhibitor cocktail]. The cells were scraped off from the petri dish using a cell scraper and transferred to an ice-cold 1.5 mL EP tube. For fully lysed, the cells were slowly rotated at 4°C for 15–30 min and then centrifuged at 12,000×g for 15 min at 4°C. The cell lysates were transferred to a new Eppendorf tube and the cell debris were discarded. Flag M2 agarose beads (Sigma) were washed with RIPA buffer for three times and then resuspended by the same volume. Every 1 mL of cell lysates were added with 40 μL pre-mixed solution of anti-FLAG M2 agarose beads and then incubated with rotation for 6–8 h at 4°C, followed by centrifugation at 3,000×g at 4°C for 5 min to obtain the immunoprecipitates. After washed three times with 1 mL ice-cold RIPA buffer, the immunoprecipitates were resuspended in 40 μL 2×loading buffer and then boiled for 10min. A part of the sample was sent to company for mass spectrometric detection and the remainder was stored at-80°C for later use. Analysis of the Mass Spectrometry data by bioinformatics.

### Immunoblot analysis and Co-IP

For immunoblot experiments, treated cells were lysed in lysis buffer (10 mM phosphate pH 7.4, 137 mM NaCl, 1% NP-40, 0.5% sodium deoxycholate, and 0.1% SDS) supplemented with 1 mM PMSF and protease inhibitor cocktail. Total or fractioned cellular extracts were mixed with 5x SDS gel loading buffer and resolved on SDS-PAGE. For Co-IP, treated cells were lysed in RIPA buffer for 30 minutes on ice. A portion of the lysate was taken as the input, and the remaining lysate was immunoprecipitated using anti-Flag antibody or anti-NCOA2 antibody. The membranes were blocked in 5% skim milk powder in TBST buffer for 1 h at room temperature and probed with the indicated primary antibodies overnight at 4°C. After hybridization with either goat anti-rabbit or goat anti-mouse secondary antibodies at a dilution of 1:5000 in TBST buffer, the membranes were washed with TBST buffer four times (10 min each) before visualization with ECL reagents (GE).

### Immunofluorescence assay

HeLa cells were plated onto coverslips in 24-well plates and transfected with the indicated plasmids. At 24 h prior to transfection, cells were washed twice with PBS and fixed in 4% paraformaldehyde for 15 min. Cells were permeabilized with 0.1% Triton X-100 for 15 min and blocked for 30 min with 1% bovine serum albumin (BSA) in PBS, followed by incubation with the specific primary antibody overnight at 4°C. After five washes with PBS containing 0.1% Tween 20, cells were incubated with FITC- or Cy3-conjugated secondary antibodies and 4′,6-DAPI for 1 h at room temperature. Finally, the coverslips were washed extensively and fixed onto slides. Slides were visualized with a DM6000B fluorescence microscope (Leica, Inc., Solms, Germany) and photographed by using a digital camera and software (Leica, Inc.). To determine the localization of endogenous RTA and NCOA2, we treated BCBL1, BC3 and JSC1 cells with VPA. At 12 h after VPA treatment, cells were fixed and stained using antibodies specific to NCOA2 and RTA.

### GST pull-down assay

GST-tagged NCOA2 fusion protein, His-tagged RTA protein and His-tagged RTA deletion (His-C544-691) protein were expressed in *Escherichia coli* BL21(DE3). The bacterial cells expressing recombinant proteins were harvested and sonicated, and proteins were solubilized in PBS with PMSF and protease inhibitor cocktail. In the pull-down assay, the bacterial lysates containing GST or GST-NCOA2 were initially incubated with 30 μl glutathione-Sepharose 4B with rotation for 4 h at 4°C. After the samples were washed, purified GST or GST-NCOA2 protein was mixed with protein lysates containing His-RTA or His-C544-691 for another 6 h at 4°C, followed by five washes in RIPA buffer. Proteins that were pulled down by glutathione beads were extracted and subjected to immunoblot analysis.

### RNA isolation and quantitative real-time PCR (RT-qPCR)

Total RNA was isolated from cells using TRIzol reagent (Invitrogen) following the manufacturer’s instructions. One microgram of RNA was used for reverse transcription with gDNA Eraser reverse transcription kits (Toyobo). cDNA was used for quantification of the indicated mRNA on a QuantStudio 6 Flex rt-PCR System (Applied Biosystems) by using SYBR Green real-time PCR master mix kits (Toyobo) according to the manufacturer’s instructions. Dissociation curve analysis of products was conducted at the end of each PCR to detect and validate the specific amplification of PCR products. Transcript levels of each gene were normalized to the GAPDH level, and the 2^−ΔΔCT^ method was used to analyze gene expression in samples. Data represent fold changes compared to the level for untreated control cells. The primers used in RT-qPCR are listed in Table 1.

### Protein stability assay

First, 293T cells were cotransfected with the indicated plasmids. At 24 h post-transfection, cells were treated with 100 μg/ml of CHX to inhibit de novo protein synthesis. At the indicated time points after treatment with CHX, the cells were harvested and subjected to immunoblot analysis. Protein levels normalized to α-Tubulin levels were quantified by densitometry.

### Ubiquitination assay

First, 293T cells were transfected with relevant expression plasmids and then treated with DMSO or MG132 (0.5 μM) (Sigma) for 12 h. The cells were harvested, lysed and immunoprecipitated using anti-Flag antibody. The precipitates were subjected to 6% SDS-PAGE followed by immunoblotting using an anti-ubiquitin rabbit antibody (ABclonal).

### Establishment and identification of stable cell lines

NCOA2-overexpressing lentiviruses were constructed based on the lentiviral vector pCDH-CMV-Flag-IRES-Blast. This NCOA2-overexpressing lentiviral vector and empty vectors were packaged in 293T cells by cotransfection with the Δ8.9 packaging plasmid and a plasmid expressing vesicular stomatitis virus G protein (pVSV-G) as described previously [60]. Two days later, the virus stock was collected and cleared by a 0.45 μm pore size filter. The NCOA2 stably expressing iSLK.RGB or BCBL1 cell line was achieved by addition of the NCOA2 stable expression and control lentiviral particles and centrifugation at 2500 rpm for 2 h. The medium was replaced by fresh DMEM. At 48 h post-infection, the cells were screened with 25 μg/ml blasticidin (Sigma), and immunoblotting was performed to determine the expression of NCOA2.

### RNA Interference

sh-NCOA2 was cloned into pLKO.1, and the target sequence of NCOA2 shRNA was 5'- ATCCGTTCTCAGACTACTAAT-3'. A control sh-GFP (target sequence: 5'-GCTACCTGTTCCATGGCCAA-3') was also cloned into pLKO.1. shRNA plasmids were transfected into 293T cells. Two siRNA oligonucleotides against NCOA2 and the corresponding negative control siRNA were obtained from GenePharma. Sequences are as follows: si#1, 5′-GGAACAGCCAUACCUUCAATT-3′; si#2, 5′-GCAGUUCUCCAGAUGACUUTT-3′. Transfection of siRNA into iSLK.RGB or BCBL1 cell line was performed by Lipofectamine 2000 according to manufacturer’s instructions.

### Generation of NCOA2-deficient iSLK.RGB cells

NCOA2-deficient iSLK.RGB cells were generated by using the CRIPR/Cas9 system as described previously [61]. The single guide RNA (sgRNA) sequence targeting human NCOA2 gene (5’-CACCGAAAATACCTCTGACCCCTCC-3’) was cloned into lentiCRISPR v2 vector and applied for production the recombined lentivirus. iSLK.RGB cells were infected with NCOA2 lentiCRISPR v2 lentivirus or the empty vector lentiCRISPR v2 lentivirus (negative control). After 48 h postinfection, blasticidin (25 μg/ml) was added to select the positive clones. Finally, the monoclonal cells acquired by using the limiting dilution method were expanded and knockout of NCOA2 was confirmed by Western blotting.

### PCR analysis of virus progeny

For analysis of whether NCOA2 can promote the production of viral progeny, 200 μl of supernatant from induced iSLK.RGB or BCBL1 stable cell line was collected and treated with DNase I (Sigma) for 1 h at 37°C. The KSHV genome was purified from the supernatant by a TIANamp Blood DNA Kit (TIANGEN) and quantified by qPCR using K9 primers for KSHV copy numbers.

### Infection of 293T cells with progeny virus

The supernatant (500 μl) of each group was incubated with 293T cells in a 12-well plate, and the plate was then centrifuged at 2500 rpm for 2 h. The medium was replaced by fresh DMEM, and the cells were cultured for an additional 24 h. The infection rate of 293T cells was examined by fluorescence microscopy based on the RFP signal.

### Flow cytometry

Cells were collected and analyzed by flow cytometry to detect KSHV lytic cells based on the EGFP signal.

### Statistical analysis

The results are expressed as the mean ± SD. Statistical analyses were performed on data from triplicate experiments by using Student’s t-test. A P value < 0.05 was considered significant, and a P value < 0.01 was considered highly significant.

## Supporting information

S1 Table Proteins interacted with RTA. (DOCX)

S1 Fig Identification proteins interacted with RTA. (A) Schematic strategy for purification and identification of RTA binding proteins via IP assay. Plasmid expressing Flag-tagged RTA was transient transfection into HEK293T or HEK293T.219 cells. The same amount of empty vector was transient transfected as a control. Cell lysates were performed to affinity purification by immunoprecipitation with FLAG M2 beads. The purified elutes were boiled in SDS-PAGE loading buffer, and then were analyzed by MS. (B) Venn diagram showing the overlaps of differentially candidate RTA binding proteins in HEK293T cells and HEK293T.219 cells.
(TIF)

S2 Fig Confocal fluorescent images of RTA truncation mutants in Hela cells. HeLa cells were transfected with RTA and all the mutants. Twenty-four hours after transfection, cells were harvested, fixed, permeabilized, and probed with anti-flag antibody. Cy3 was used to visualize the stained truncation proteins. Diamidino-2-phenylindole shows the nuclei of cells.
(TIF)

S3 Fig Effect of NCOA2 and vSP1 on RTA expression. (A) 293T cells were transfected with the indicated expression plasmids. The expression of RTA protein was examined by immunoblotting with the indicated antibodies. (B) 293T cells were cotransfected with HA-RTA and Myc-NCOA2 together with an increasing amount of Flag-vSP1 (0, 0.5, 1, 2 μg) for 36 h. Cell lysates were collected and subjected to western blotting with the indicated antibodies. (C) 293T cells were cotransfected with HA-RTA and Flag-vSP1 together with an increasing amount of Myc-NCOA2 (0, 0.5, 1, 2 μg) for 36 h. Cell lysates were collected and subjected to western blotting with the indicated antibodies.
(TIF)

S4 Fig Overexpression of NCOA2 enhances KSHV lytic replication. (A) The supernatants (500 μl) from dox-induced iSLK.RGB-Vector and iSLK.RGB-NCOA2 cells at 48 hpi were incubated with 293T cells. The infection rate of 293T cells was examined by fluorescence microscopy. (B) BCBL1-NCOA2 and BCBL1-Vector cells were treated with VPA for 24 h, and the transcription of viral genes was analyzed by qPCR with the indicated primers. Data were pooled from three independent experiments and were analyzed with a two-tailed Student’s *t*-test (****P < 0.0001). (C) NCOA2 overexpression increases the expression of viral genes in BCBL1 cells. BCBL1-NCOA2 and BCBL1-Vector cells were treated by VPA for 24 h, the expression level of RTA protein and ORF64 protein was determined by immunoblotting with the indicated antibodies.
(TIF)

S5 Fig Knockout of endogenous NCOA2 impairs viral lytic replication. (A) Generation of NCOA2-deficient iSLK.RGB cell line. NCOA2-deficient iSLK.RGB cells were generated by using the CRISPR/Cas9 system. NCOA2 knockout was confirmed by western blotting with anti-NCOA2 rabbit antibody. NCOA2-deficient iSLK.RGB cells (NCOA2^-/-^) or wild-type iSLK.RGB cells (WT) were treated with dox for 48 h, the expression levels of RTA and ORF64 were examined by western blotting. (B) The progeny viruses form culture supernatants were quantified by qPCR. Data were pooled from three independent experiments and were analyzed with a two-tailed Student’s *t*-test (**P < 0.01).
(TIF)
