# Supplementary material for: NCOA2 promotes lytic reactivation of Kaposi’s sarcoma-associated herpesvirus by enhancing the expression of the master switch protein RTA
Source: PLoS Pathog. 2019 Nov 21;15(11):e1008160. doi: 10.1371/journal.ppat.1008160 (PMC6894885; doi:10.1371/journal.ppat.1008160)
Supplement: S1 Table — (DOCX) [file ppat.1008160.s001.docx]

| **Uniport Number** | **Protein Function** |
| --- | --- |
| A0A140VK53 | HUMAN Testicular secretory protein Li 53 OS=Homo sapiens PE=2 SV=1 |
| A0A024RD80 | HUMAN Heat shock protein 90kDa alpha (Cytosolic), class B member 1, isoform CRA_a OS=Homo sapiens GN=HSP90AB1 PE=3 SV=1 |
| A0A087WTT1 | HUMAN Polyadenylate-binding protein OS=Homo sapiens GN=PABPC1 PE=1 SV=1 |
| P11021 | HUMAN 78 kDa glucose-regulated protein OS=Homo sapiens GN=HSPA5 PE=1 SV=2 |
| A8K4W0 | HUMAN 40S ribosomal protein S3a OS=Homo sapiens GN=RPS3A PE=2 SV=1 |
| Q5EC54 | HUMAN Heterogeneous nuclear ribonucleoprotein K transcript variant OS=Homo sapiens GN=HNRPK PE=2 SV=1 |
| J3KTA4 | HUMAN Probable ATP-dependent RNA helicase DDX5 OS=Homo sapiens GN=DDX5 PE=1 SV=1 |
| Q12905 | HUMAN Interleukin enhancer-binding factor 2 OS=Homo sapiens GN=ILF2 PE=1 SV=2 |
| A0A024RDF4 | HUMAN Heterogeneous nuclear ribonucleoprotein D (AU-rich element RNA binding protein 1, 37kDa), isoform CRA_e OS=Homo sapiens GN=HNRPD PE=4 SV=1 |
| A2A3R6 | HUMAN 40S ribosomal protein S6 OS=Homo sapiens GN=RPS6 PE=2 SV=1 |
| E9PI39 | HUMAN Elongation factor 1-delta (Fragment) OS=Homo sapiens GN=EEF1D PE=1 SV=1 |
| P50991 | HUMAN T-complex protein 1 subunit delta OS=Homo sapiens GN=CCT4 PE=1 SV=4 |
| P62249 | HUMAN 40S ribosomal protein S16 OS=Homo sapiens GN=RPS16 PE=1 SV=2 |
| A0A024R1V4 | HUMAN 60S ribosomal protein L27 OS=Homo sapiens GN=RPL27 PE=3 SV=1 |
| P27708 | HUMAN CAD protein OS=Homo sapiens GN=CAD PE=1 SV=3 |
| Q53HV2 | HUMAN Chaperonin containing TCP1, subunit 7 (Eta) variant (Fragment) OS=Homo sapiens PE=2 SV=1 |
| Q16629 | HUMAN Serine/arginine-rich splicing factor 7 OS=Homo sapiens GN=SRSF7 PE=1 SV=1 |
| Q01130 | HUMAN Serine/arginine-rich splicing factor 2 OS=Homo sapiens GN=SRSF2 PE=1 SV=4 |
| Q6IAX2 | HUMAN RPL21 protein OS=Homo sapiens GN=RPL21 PE=2 SV=1 |
| Q9NX58 | HUMAN Cell growth-regulating nucleolar protein OS=Homo sapiens GN=LYAR PE=1 SV=2 |
| F5GY37 | HUMAN Prohibitin-2 OS=Homo sapiens GN=PHB2 PE=1 SV=1 |
| Q14498 | HUMAN RNA-binding protein 39 OS=Homo sapiens GN=RBM39 PE=1 SV=2 |
| A8K4T2 | HUMAN cDNA FLJ76281, highly similar to Homo sapiens DnaJ (Hsp40) homolog, subfamily C, member 7 (DNAJC7), mRNA OS=Homo sapiens PE=2 SV=1 |
| P84090 | HUMAN Enhancer of rudimentary homolog OS=Homo sapiens GN=ERH PE=1 SV=1 |
| Q6FI35 | HUMAN Proliferating cell nuclear antigen OS=Homo sapiens GN=PCNA PE=1 SV=1 |
| P83881 | HUMAN 60S ribosomal protein L36a OS=Homo sapiens GN=RPL36A PE=1 SV=2 |
| Q15596 | HUMAN Nuclear receptor coactivator 2 OS=Homo sapiens GN=NCOA2 PE=1 SV=2 |
| Q99613 | HUMAN Eukaryotic translation initiation factor 3 subunit C OS=Homo sapiens GN=EIF3C PE=1 SV=1 |
| Q01780 | HUMAN Exosome component 10 OS=Homo sapiens GN=EXOSC10 PE=1 SV=2 |
| A0A0F7NGI8 | HUMAN Leucine rich repeat (In FLII) interacting protein 1, isoform CRA_c OS=Homo sapiens GN=LRRFIP1 PE=2 SV=1 |
| B4DM85 | HUMAN Kinesin-like protein OS=Homo sapiens PE=2 SV=1 |
| P32969 | HUMAN 60S ribosomal protein L9 OS=Homo sapiens GN=RPL9 PE=1 SV=1 |
| P42677 | HUMAN 40S ribosomal protein S27 OS=Homo sapiens GN=RPS27 PE=1 SV=3 |
| O43167 | HUMAN Zinc finger and BTB domain-containing protein 24 OS=Homo sapiens GN=ZBTB24 PE=1 SV=2 |
| P37108 | HUMAN Signal recognition particle 14 kDa protein OS=Homo sapiens GN=SRP14 PE=1 SV=2 |
| P31153 | HUMAN S-adenosylmethionine synthase isoform type-2 OS=Homo sapiens GN=MAT2A PE=1 SV=1 |
| Q08J23 | HUMAN tRNA (cytosine(34)-C(5))-methyltransferase OS=Homo sapiens GN=NSUN2 PE=1 SV=2 |
| Q15057 | HUMAN Arf-GAP with coiled-coil, ANK repeat and PH domain-containing protein 2 OS=Homo sapiens GN=ACAP2 PE=1 SV=3 |
| Q5C9Z4 | HUMAN Nucleolar MIF4G domain-containing protein 1 OS=Homo sapiens GN=NOM1 PE=1 SV=1 |
| A4FU77 | HUMAN SNRNP200 protein (Fragment) OS=Homo sapiens GN=SNRNP200 PE=2 SV=1 |
| B3KQV6 | HUMAN Serine/threonine-protein phosphatase 2A 65 kDa regulatory subunit A alpha isoform OS=Homo sapiens GN=PPP2R1A PE=1 SV=1 |
| H0Y4R1 | HUMAN Inosine-5'-monophosphate dehydrogenase 2 (Fragment) OS=Homo sapiens GN=IMPDH2 PE=1 SV=1 |
| K7ES00 | HUMAN Histone H3.3 (Fragment) OS=Homo sapiens GN=H3F3B PE=1 SV=1 |
| Q05DF2 | HUMAN SF3A2 protein (Fragment) OS=Homo sapiens GN=SF3A2 PE=2 SV=1 |
| Q9HBB9 | HUMAN HC56 OS=Homo sapiens PE=4 SV=1 |
| A0A075B6Z2 | HUMAN T-cell receptor alpha joining 56 (Fragment) OS=Homo sapiens GN=TRAJ56 PE=4 SV=1 |
| Q53XC0 | HUMAN Eukaryotic translation initiation factor 2, subunit 1 alpha, 35kDa, isoform CRA_a OS=Homo sapiens GN=EIF2S1 PE=2 SV=1 |
| O95684 | HUMAN FGFR1 oncogene partner OS=Homo sapiens GN=FGFR1OP PE=1 SV=1 |
| P09661 | HUMAN U2 small nuclear ribonucleoprotein A' OS=Homo sapiens GN=SNRPA1 PE=1 SV=2 |
| P62304 | HUMAN Small nuclear ribonucleoprotein E OS=Homo sapiens GN=SNRPE PE=1 SV=1 |
| P63241 | HUMAN Eukaryotic translation initiation factor 5A-1 OS=Homo sapiens GN=EIF5A PE=1 SV=2 |
| Q14444 | HUMAN Caprin-1 OS=Homo sapiens GN=CAPRIN1 PE=1 SV=2 |
| Q8TCT9 | HUMAN Minor histocompatibility antigen H13 OS=Homo sapiens GN=HM13 PE=1 SV=1 |
| Q96A05 | HUMAN V-type proton ATPase subunit E 2 OS=Homo sapiens GN=ATP6V1E2 PE=1 SV=1 |
| Q96CS3 | HUMAN FAS-associated factor 2 OS=Homo sapiens GN=FAF2 PE=1 SV=2 |
| Q9BYN8 | HUMAN 28S ribosomal protein S26, mitochondrial OS=Homo sapiens GN=MRPS26 PE=1 SV=1 |
| Q9P013 | HUMAN Spliceosome-associated protein CWC15 homolog OS=Homo sapiens GN=CWC15 PE=1 SV=2 |
| Q9UNM6 | HUMAN 26S proteasome non-ATPase regulatory subunit 13 OS=Homo sapiens GN=PSMD13 PE=1 SV=2 |
| A0A024R9K4 | HUMAN Forty-two-three domain containing 1, isoform CRA_a OS=Homo sapiens GN=FYTTD1 PE=4 SV=1 |
| A0A024RDT4 | HUMAN Lymphocyte cytosolic protein 1 (L-plastin), isoform CRA_a OS=Homo sapiens GN=LCP1 PE=4 SV=1 |
| A0A087X1B7 | HUMAN Chromatin target of PRMT1 protein OS=Homo sapiens GN=CHTOP PE=1 SV=1 |
| A0A0A0MRN4 | HUMAN DBIRD complex subunit ZNF326 OS=Homo sapiens GN=ZNF326 PE=1 SV=1 |
| A0A0B4J2E5 | HUMAN Uncharacterized protein OS=Homo sapiens GN=LOC102724159 PE=1 SV=1 |
| A0A1W2PRL9 | HUMAN Transcription elongation factor A protein 1 OS=Homo sapiens GN=TCEA1 PE=1 SV=1 |
| A8K201 | HUMAN cDNA FLJ75605, highly similar to Homo sapiens CGI-115 protein (CGI-115), mRNA OS=Homo sapiens PE=2 SV=1 |
| B3KPV5 | HUMAN cDNA FLJ32300 fis, clone PROST2002227, highly similar to U3 small nucleolar ribonucleoprotein protein MPP10 (Fragment) OS=Homo sapiens PE=2 SV=1 |
| B3KPY4 | HUMAN Zinc finger protein 498, isoform CRA_a OS=Homo sapiens GN=ZNF498 PE=2 SV=1 |
| B4DNH1 | HUMAN Profilin OS=Homo sapiens PE=2 SV=1 |
| B4DYG5 | HUMAN cDNA FLJ60960, highly similar to Phosphatidylinositol 4-kinase alpha (EC 2.7.1.67) OS=Homo sapiens PE=2 SV=1 |
| B4DZV7 | HUMAN cDNA FLJ54209 OS=Homo sapiens PE=2 SV=1 |
| B5BUB1 | HUMAN RuvB-like helicase (Fragment) OS=Homo sapiens GN=RUVBL1 PE=2 SV=1 |
| D2KZ55 | HUMAN MHC class I antigen (Fragment) OS=Homo sapiens GN=HLA-A PE=3 SV=1 |
| H0YJP0 | HUMAN E3 ubiquitin-protein ligase HECTD1 (Fragment) OS=Homo sapiens GN=HECTD1 PE=1 SV=2 |
| K7EK36 | HUMAN Zinc finger protein 177 OS=Homo sapiens GN=ZNF177 PE=4 SV=1 |
| Q5CZH6 | HUMAN V-type proton ATPase subunit a OS=Homo sapiens GN=DKFZp686N0561 PE=2 SV=1 |
| Q96DP6 | HUMAN cDNA FLJ31052 fis, clone HSYRA2000629, weakly similar to SKIN SECRETORY PROTEIN XP2 OS=Homo sapiens PE=2 SV=1 |
