# Supplementary figures and images for: NCOA2 promotes lytic reactivation of Kaposi’s sarcoma-associated herpesvirus by enhancing the expression of the master switch protein RTA
Source: PLoS Pathog. 2019 Nov 21;15(11):e1008160. doi: 10.1371/journal.ppat.1008160 (PMC6894885; doi:10.1371/journal.ppat.1008160)

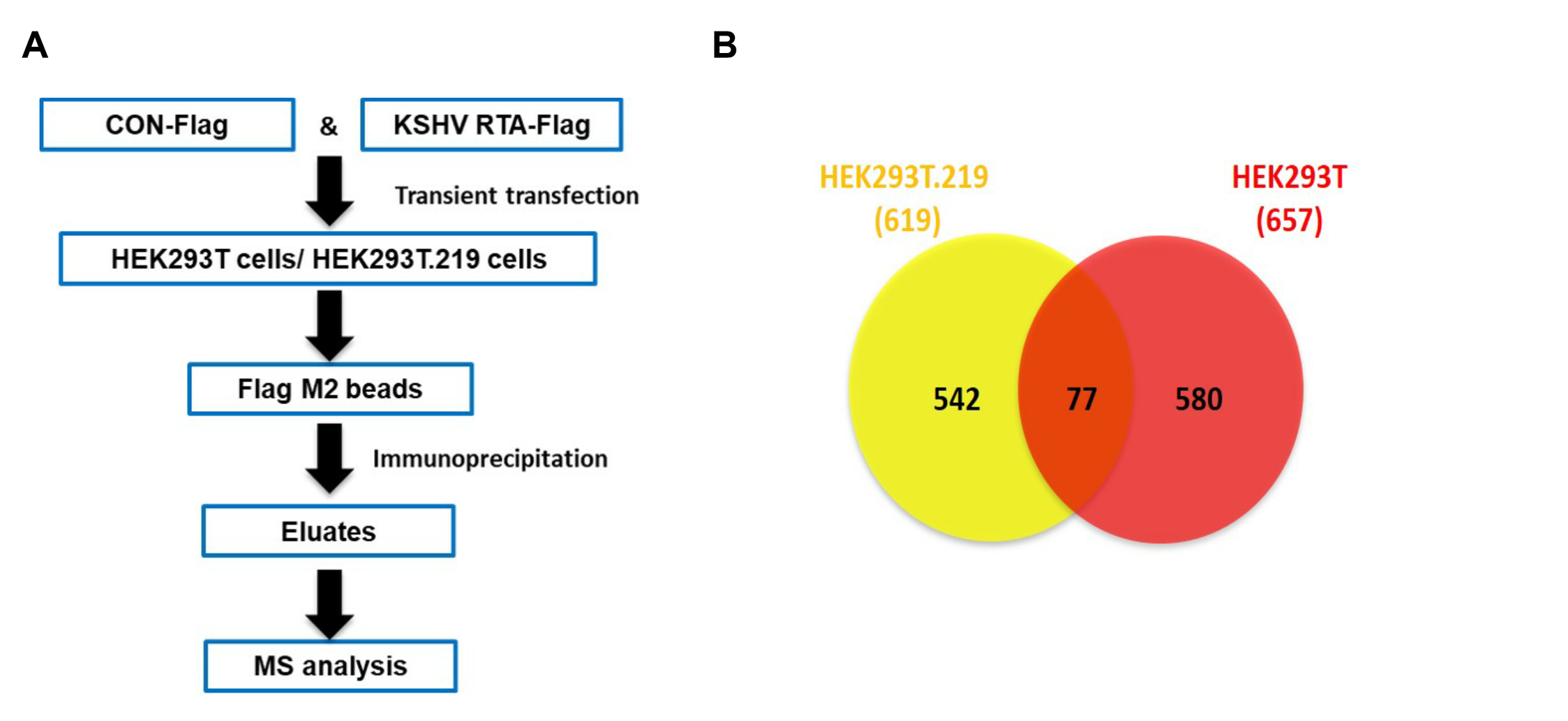

Supplement: S1 Fig — (A) Schematic strategy for purification and identification of RTA binding proteins via IP assay. Plasmid expressing Flag-tagged RTA was transient transfection into HEK293T or HEK293T.219 cells. The same amount of empty vector was transient transfected as a control. Cell lysates were performed to affinity purification by immunoprecipitation with FLAG M2 beads. The purified elutes were boiled in SDS-PAGE loading buffer, and then were analyzed by MS. (B) Venn diagram showing the overlaps of differentially candidate RTA binding proteins in HEK293T cells and HEK293T.219 cells. (TIF) [file ppat.1008160.s002.tif]

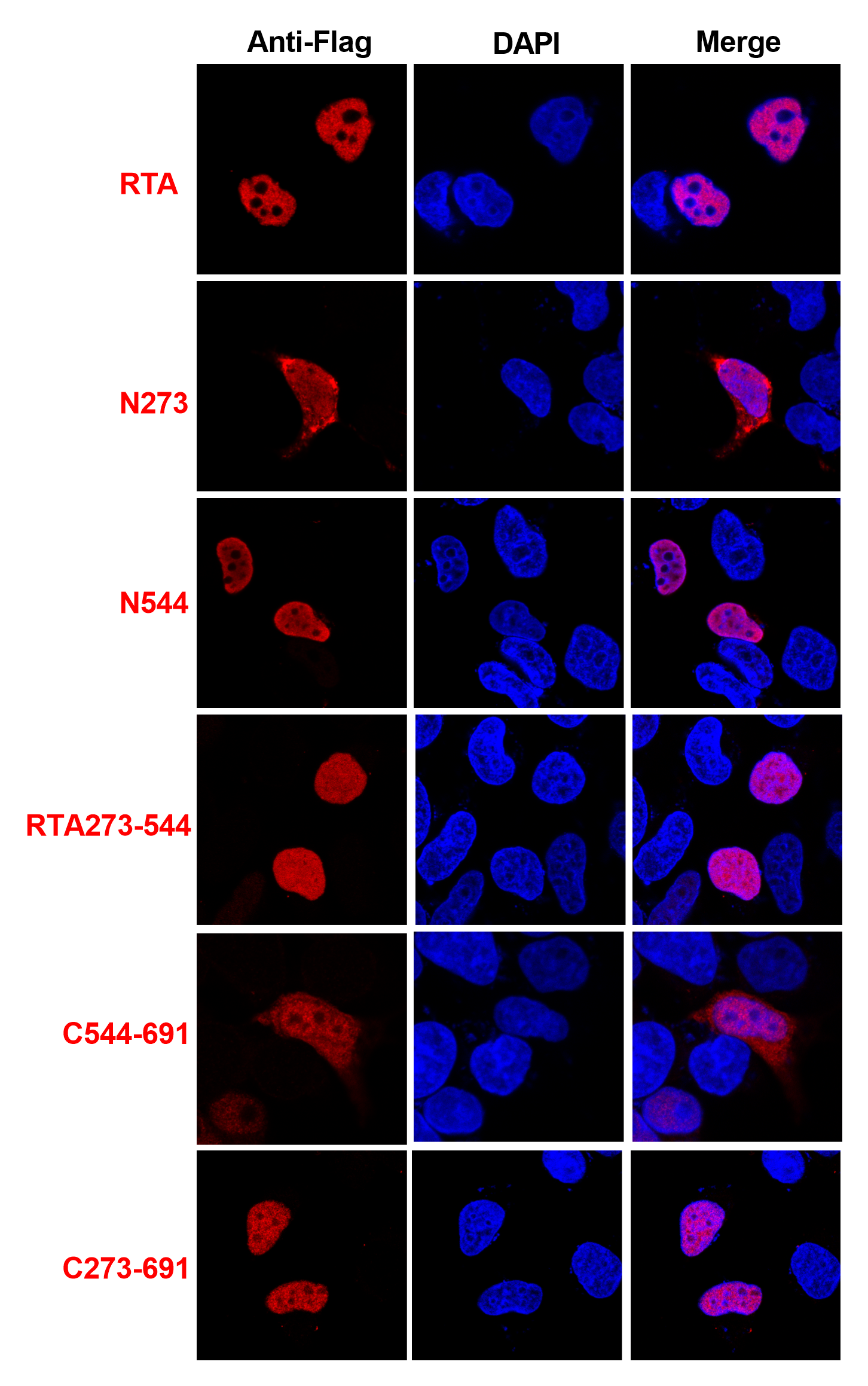

Supplement: S2 Fig — HeLa cells were transfected with RTA and all the mutants. Twenty-four hours after transfection, cells were harvested, fixed, permeabilized, and probed with anti-flag antibody. Cy3 was used to visualize the stained truncation proteins. Diamidino-2-phenylindole shows the nuclei of cells. (TIF) [file ppat.1008160.s003.tif]

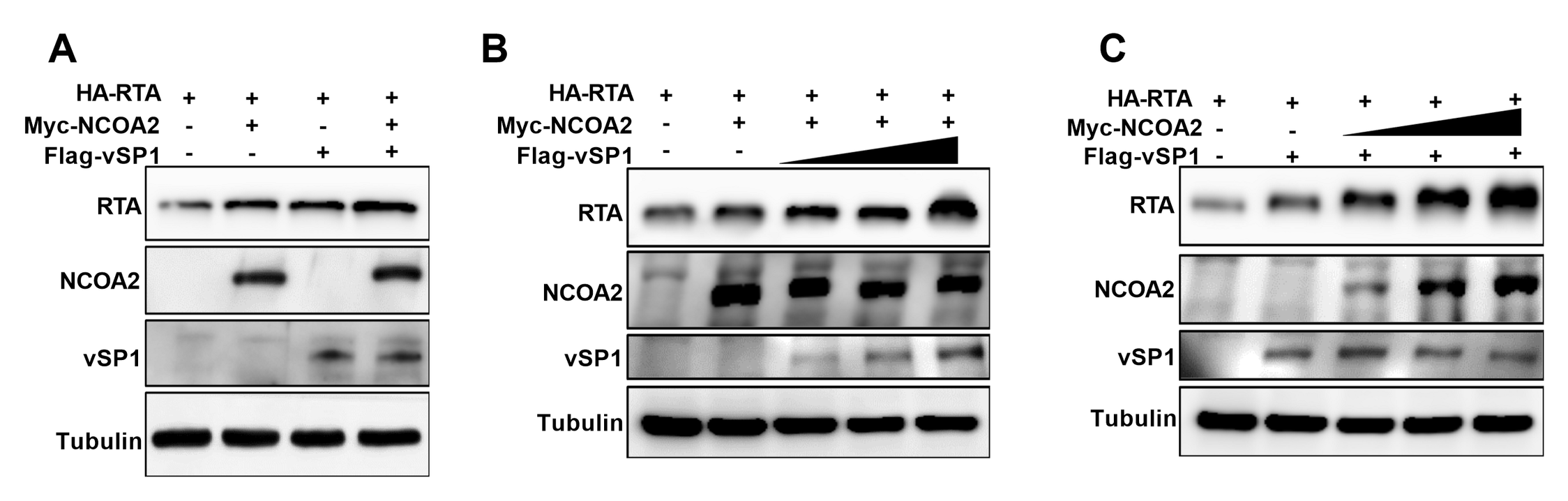

Supplement: S3 Fig — (A) 293T cells were transfected with the indicated expression plasmids. The expression of RTA protein was examined by immunoblotting with the indicated antibodies. (B) 293T cells were cotransfected with HA-RTA and Myc-NCOA2 together with an increasing amount of Flag-vSP1 (0, 0.5, 1, 2 μg) for 36 h. Cell lysates were collected and subjected to western blotting with the indicated antibodies. (C) 293T cells were cotransfected with HA-RTA and Flag-vSP1 together with an increasing amount of Myc-NCOA2 (0, 0.5, 1, 2 μg) for 36 h. Cell lysates were collected and subjected to western blotting with the indicated antibodies. (TIF) [file ppat.1008160.s004.tif]

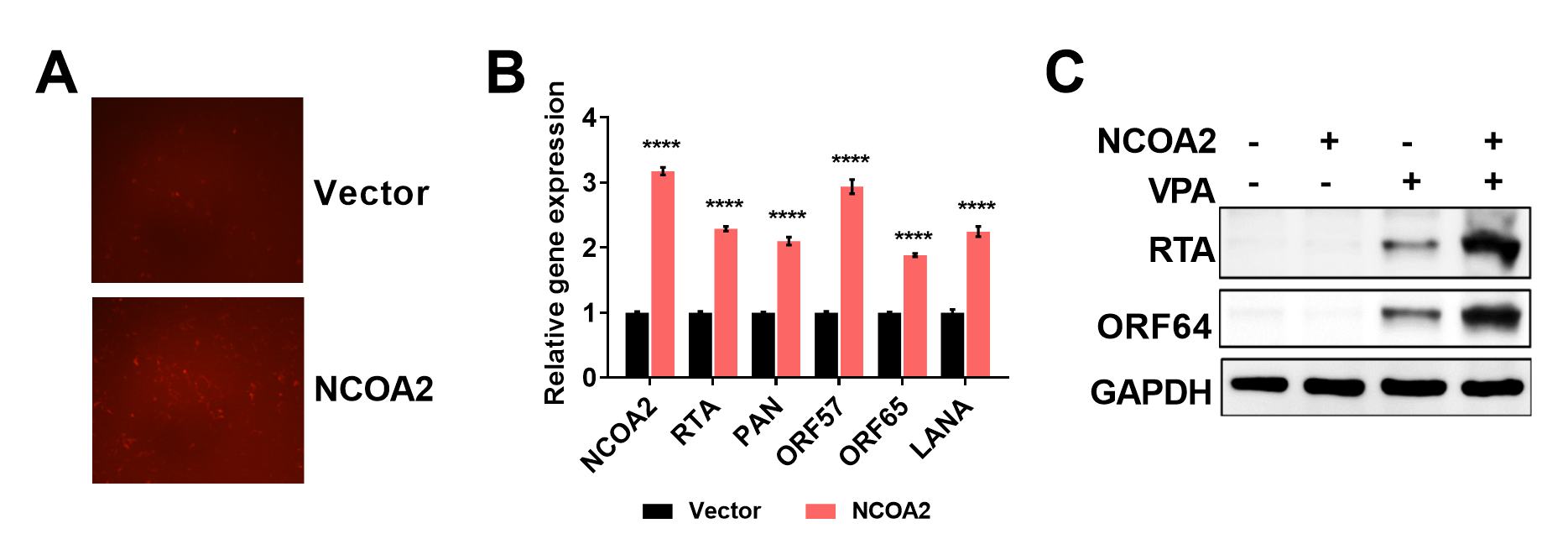

Supplement: S4 Fig — (A) The supernatants (500 μl) from dox-induced iSLK.RGB-Vector and iSLK.RGB-NCOA2 cells at 48 hpi were incubated with 293T cells. The infection rate of 293T cells was examined by fluorescence microscopy. (B) BCBL1-NCOA2 and BCBL1-Vector cells were treated with VPA for 24 h, and the transcription of viral genes was analyzed by qPCR with the indicated primers. Data were pooled from three independent experiments and were analyzed with a two-tailed Student’s t-test (****P < 0.0001). (C) NCOA2 overexpression increases the expression of viral genes in BCBL1 cells. BCBL1-NCOA2 and BCBL1-Vector cells were treated by VPA for 24 h, the expression level of RTA protein and ORF64 protein was determined by immunoblotting with the indicated antibodies. (TIF) [file ppat.1008160.s005.tif]

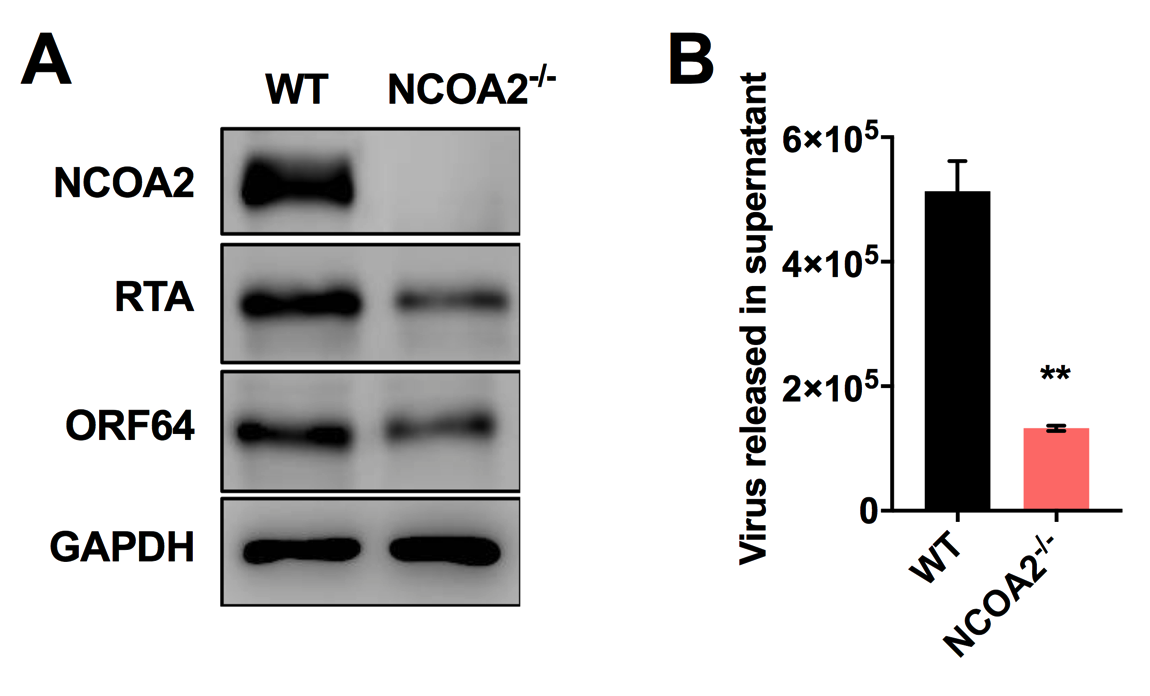

Supplement: S5 Fig — (A) Generation of NCOA2-deficient iSLK.RGB cell line. NCOA2-deficient iSLK.RGB cells were generated by using the CRISPR/Cas9 system. NCOA2 knockout was confirmed by western blotting with anti-NCOA2 rabbit antibody. NCOA2-deficient iSLK.RGB cells (NCOA2-/-) or wild-type iSLK.RGB cells (WT) were treated with dox for 48 h, the expression levels of RTA and ORF64 were examined by western blotting. (B) The progeny viruses form culture supernatants were quantified by qPCR. Data were pooled from three independent experiments and were analyzed with a two-tailed Student’s t-test (**P < 0.01). (TIF) [file ppat.1008160.s006.tif]
